# Supplementary material for: Terrestrial reproduction and parental care drive rapid evolution in the trade-off between offspring size and number across amphibians
Source: PLoS Biol. 2022 Jan 4;20(1):e3001495. doi: 10.1371/journal.pbio.3001495 (PMC8726499; doi:10.1371/journal.pbio.3001495)
Supplement: S1 Table — Here, we only report results of variables of interest to the aims of our study. (DOCX) [file pbio.3001495.s001.docx]

**S1 Table. Summary of previous phylogenetic comparative studies on the correlated evolution between parental care, offspring developmental environment and/or offspring size-number tradeoff in amphibians.** Here we only report results of variables of interest to the aims of our study.

| **Study** | **N species** | **Methods** | **Results & Conclusions** | **Notes** |
| --- | --- | --- | --- | --- |
| Summers et al (2006) | 383 Anura (47 contrasts) | - Phylogenetic pairwise comparison & Discrete models in maximum likelihood - Supertree phylogeny with equal branch lengths | - Parental care strongly associated with increased egg size - Egg size is unrelated to adult body size - Parental care evolves after larger eggs | - No control for allometry and clutch size on egg size - All care forms combined into a binary presence/absence variable |
| Summers et al (2007) | 383 Anura (7 to 35 contrasts) | - Phylogenetic pairwise comparisons - Supertree phylogeny with equal branch lengths | - Egg size is greater in species with parental care and direct development - Egg size is unrelated to adult body size | - No control for allometry and clutch size on egg size - All care forms combined into a binary presence/absence variable - Relationship between egg size and each care or ecology variable tested in isolation of others |
| Gomez-Mestre et al. (2012) | 720 Anura of which 470 have life history data | - PGLS, phylogenetic ANOVA & BayesTraits Discrete models in maximum likelihood - Time calibrated molecular phylogeny | - Egg size and clutch size are negatively associated - Both egg size and clutch size increase with body size - Eggs are larger and clutches smaller in species with terrestrial reproduction than in those with aquatic reproduction - Parental care and terrestrial reproduction are strongly associated - Parental care and direct development are strongly associated | - Relationship between life history traits, and between each life history trait and reproductive ecology, are tested in isolation of each other, without accounting for co-variation between them - All care forms combined into a binary presence/absence variable - Terrestrial reproduction is not divided by developmental stage (i.e. terrestriality at egg and/or tadpole stage are both classed as terrestrial) |
| Monroe et al. (2015) | 41 to 66 Anura | - PGLS and ANCOVA - Time calibrated molecular phylogeny | - Egg size, clutch size, and total female egg investment (egg size x clutch size) increase with adult body size - No association between clutch size or egg size and parental care - Total female egg investment is lower in species with parental care | - Associations between life history traits, and between each life history trait and parental care, are tested in isolation of each other, i.e. without accounting for co-variation between them - All care forms combined into a binary presence/absence variable |
| Vagi et al. (2019) | 1044 Anura species but only 193 to 197 used in analyses with life history traits and ecology | - PGLS - Time-calibrated consensus tree containing species placed with and without molecular data | - Male care duration is longer and male degree of protection greater in species with terrestrial reproduction - Female degree of protection is longer in species with terrestrial reproduction - Female nourishment is higher in species with lower clutch volume (egg size x clutch size) and larger body size | - Parental care forms are ranked for duration, protection and nourishment, merging different behaviours and diverse care forms at different stages of offspring development - Ranked parental care variables are treated as continuous dependent variable in PGLS models, potentially violating assumptions of linear models - Clutch volume does not allow discrimination of effects related to changes in egg size vs those in clutch size - Terrestrial reproduction is not divided by developmental stage (i.e. terrestriality at egg and/or tadpole stage are both classed as terrestrial) - Use of phylogenies with species added based on taxonomy can lead to misleading results (Rabosky 2015) |
| Vagi et al. (2020) | 971 Anura species but 126 to 383 used in analyses with care and reproductive ecology | - Phylogenetic GLM models - Time-calibrated consensus tree containing species placed with and without molecular data | - Female attendance and male attendance are associated with terrestrial reproduction - Carrying by males or females is unrelated to terrestrial reproduction - Nourishment by females (endotrophy) is unrelated to terrestrial reproduction - Trophic egg feeding is unrelated to terrestrial reproduction | - Forms of care (i.e. nest building, attendance, carrying, endotrophy, and trophic egg feeding) not clearly defined; ‘carrying’ conflates transport, brooding, and viviparity; forms of care do not account for the stage of offspring development at which they occur (i.e. egg, tadpole, juvenile) - Terrestrial reproduction is not divided by developmental stage (i.e. terrestriality at egg and/or tadpole stage are both classed as terrestrial) - Environmental and social correlates of each form of care are tested in isolation of all other forms of care, although they are later shown to themselves be correlated - Use of phylogenies with species added based on taxonomy can lead to misleading results (Rabosky 2015) |

**Supplementary References**

Gomez‐Mestre, I., Pyron, R. A. & Wiens, J. J. Phylogenetic analyses reveal unexpected patterns in the evolution of reproductive modes in frogs. Evolution 66, 3687-3700 (2012).

Monroe, M. J., South, S. H. & Alonzo, S. H. The evolution of fecundity is associated with female body size but not female‐biased sexual size dimorphism among frogs. Journal of Evolutionary Biology 28, 1793-1803 (2015).

Rabosky, D. L. No substitute for real data: a cautionary note on the use of phylogenies from birth–death polytomy resolvers for downstream comparative analyses. Evolution 69, 3207-3216 (2015).

Summers, K., McKeon, C. S. & Heying, H. The evolution of parental care and egg size: a comparative analysis in frogs. Proceedings of the Royal Society B 273, 687-692 (2006).

Summers, K., McKeon, C. S., Heying, H., Hall, J. & Patrick, W. Social and environmental influences on egg size evolution in frogs. Journal of Zoology 271, 225-232 (2007).

Vági, B., Végvári, Z., Liker, A., Freckleton, R. P. & Székely, T. Parental care and the evolution of terrestriality in frogs. Proceedings of the Royal Society B 286, 20182737 (2019).

Vági, B., Végvári, Z., Liker, A., Freckleton, R. P. & Székely, T. Climate and mating systems as drivers of global diversity of parental care in frogs. Global Ecology and Biogeography 29, 1373-1386 (2020).
