# Supplementary material for: Terrestrial reproduction and parental care drive rapid evolution in the trade-off between offspring size and number across amphibians
Source: PLoS Biol. 2022 Jan 4;20(1):e3001495. doi: 10.1371/journal.pbio.3001495 (PMC8726499; doi:10.1371/journal.pbio.3001495)
Supplement: S2 Table — The total sample size of our data set is 805 species, with complete parental care, offspring habitat, direct development, and life history data (body size, egg size, and clutch size). Here, we report the sample sizes for these 805 species for all predictors considered in this study, those in which more than 5 species exhibited the trait of interest. (DOCX) [file pbio.3001495.s002.docx]

S2 Table. Sample sizes for categorical, binary, independent variables considered as predictors of egg and clutch size in this study. The total sample size of our dataset is 805 species, with complete parental care, offspring habitat, direct development, and life history data (body size, egg size and clutch size). Here we report the sample sizes for these 805 species for all predictors considered in this study, those in which more than 5 species exhibited the trait of interest.

| **Variable** | **Without** | **With** |
| --- | --- | --- |
| Egg attendance (male) | 712 | 93 |
| Egg attendance (female) | 706 | 99 |
| Egg brooding | 787 | 18 |
| Tadpole attendance (male) | 796 | 9 |
| Tadpole attendance (female) | 798 | 7 |
| Tadpole transport (male) | 775 | 30 |
| Tadpole transport (female) | 799 | 6 |
| Tadpole brooding | 799 | 6 |
| Tadpole feeding | 794 | 11 |
| Juvenile attendance | 797 | 8 |
| Viviparity | 799 | 6 |
| Terrestrial eggs | 529 | 276 |
| Terrestrial larvae | 785 | 20 |
| Direct development | 713 | 92 |
