# Supplementary material for: Terrestrial reproduction and parental care drive rapid evolution in the trade-off between offspring size and number across amphibians
Source: PLoS Biol. 2022 Jan 4;20(1):e3001495. doi: 10.1371/journal.pbio.3001495 (PMC8726499; doi:10.1371/journal.pbio.3001495)
Supplement: S3 Table — Results for the analysis of egg size in (A) full model, including all predictor variables, and (B) the simplest statistically justifiable model (reduced model) with only significant predictors after model simplification (see Methods, Identifying significant predictors of egg and clutch size evolution). Results of analysis for clutch size in (C) full model and (D) reduced model. The columns report the ESS, the mean and median of the posterior distributions, the 95% HPD interval, and the proportion of the posterior distribution crossing zero (Px) for each predictor variable in the model. We also report model R2, phylogenetic signal as estimated by λ, and model marginal likelihood. ESS, effective sample size; HPD, highest posterior density. (DOCX) [file pbio.3001495.s003.docx]

**S3 Table. Variable rates models for egg and clutch size.** Results for the analysis of egg size in (A) full model including all predictor variables, and (B) the simplest statistically justifiable model (reduced model) with only significant predictors after model simplification (see *Methods, Identifying significant predictors of egg and clutch size evolution*). Results of analysis for clutch size in (C) full model and (D) reduced model. The columns report the effective sample size (ESS), the mean and median of the posterior distributions, the 95% highest posterior density (HPD) interval, and the proportion of the posterior distribution crossing zero (P_x_) for each predictor variable in the model. We also report model R^2^, phylogenetic signal as estimated by λ, and model marginal likelihood.

| 1. **Egg size: Full model** | | | | | |
| --- | --- | --- | --- | --- | --- |
| **Parameter** | **ESS** | **Mean** | **Median** | **95% HPD** | **P_x_** |
| Intercept | 1885 | -0.164 | -0.162 | [-0.2728, -0.0471] | 0.004 |
| Clutch size | 2000 | -0.117 | -0.117 | [-0.1371, -0.0957] | 0.000 |
| Body size | 1874 | 0.391 | 0.390 | [0.3365, 0.4492] | 0.000 |
| Egg attendance (male) | 2000 | 0.088 | 0.089 | [0.0428, 0.1304] | 0.000 |
| Egg attendance (female) | 2000 | 0.053 | 0.053 | [0.0031, 0.1058] | 0.028 |
| Egg brooding | 2000 | 0.178 | 0.179 | [0.0815, 0.2843] | 0.002 |
| Tadpole attendance (male) | 2000 | -0.056 | -0.055 | [-0.1418, 0.0239] | 0.095 |
| Tadpole attendance (female) | 2000 | -0.269 | -0.264 | [-0.4438, -0.1019] | 0.001 |
| Tadpole transport (male) | 2000 | -0.043 | -0.043 | [-0.1439, 0.0521] | 0.198 |
| Tadpole transport (female) | 2000 | -0.045 | -0.049 | [-0.1789, 0.1067] | 0.262 |
| Tadpole brooding | 2000 | 0.097 | 0.096 | [-0.0479, 0.2394] | 0.101 |
| Tadpole feeding | 2000 | -0.153 | -0.154 | [-0.2420, -0.0634] | 0.002 |
| Juvenile attendance | 2000 | -0.021 | -0.020 | [-0.1232, 0.0788] | 0.358 |
| Viviparity | 2000 | -0.134 | -0.136 | [-0.2931, 0.0191] | 0.049 |
| Terrestrial eggs | 1841 | 0.078 | 0.077 | [0.0403, 0.1128] | 0.000 |
| Terrestrial larvae | 2000 | -0.011 | -0.011 | [-0.0807, 0.0689] | 0.385 |
| Direct development | 2000 | 0.157 | 0.157 | [0.1033, 0.2151] | 0.000 |
| R^2^ | 2000 | 0.461 | 0.461 | [0.4068, 0.5096] | NA |
| λ | 2000 | 0.786 | 0.790 | [0.6887, 0.8761] | NA |
| Model likelihood | 2000 | 592.900 | 592.900 | [572.8075, 611.6302] | NA |

| 1. **Egg size: Reduced model** | | | | | |
| --- | --- | --- | --- | --- | --- |
| **Parameter** | **ESS** | **Mean** | **Median** | **95% HPD** | **P_x_** |
| Intercept | 2000 | -0.173 | -0.173 | [-0.2847, -0.0647] | 0.001 |
| Clutch size | 2000 | -0.115 | -0.115 | [-0.1342, -0.0943] | 0.000 |
| Body size | 2000 | 0.392 | 0.393 | [0.3396, 0.4508] | 0.000 |
| Egg attendance (male) | 2000 | 0.079 | 0.079 | [0.0394, 0.1180] | 0.000 |
| Egg attendance (female) | 2000 | 0.062 | 0.062 | [0.0149, 0.1111] | 0.009 |
| Egg brooding | 2000 | 0.197 | 0.196 | [0.1085, 0.2975] | 0.000 |
| Tadpole attendance (female) | 2000 | -0.298 | -0.292 | [-0.4850, -0.1295] | 0.001 |
| Tadpole feeding | 2000 | -0.166 | -0.166 | [-0.2576, -0.0863] | 0.000 |
| Terrestrial eggs | 2000 | 0.076 | 0.076 | [0.0442, 0.1135] | 0.000 |
| Direct development | 2000 | 0.143 | 0.142 | [0.0935, 0.1931] | 0.000 |
| R^2^ | 2000 | 0.455 | 0.455 | [0.4066, 0.5044] | NA |
| λ | 1841 | 0.794 | 0.799 | [0.7019, 0.8752] | NA |
| Model likelihood | 2000 | 590.600 | 590.700 | [573.2661, 608.1157] | NA |

| 1. **Clutch size: Full model** | | | | | |
| --- | --- | --- | --- | --- | --- |
| **Parameter** | **ESS** | **Mean** | **Median** | **95% HPD** | **P_x_** |
| Intercept | 2000 | -1.436 | -1.450 | [-2.0186, -0.9621] | 0.001 |
| Egg size | 1479 | -1.168 | -1.168 | [-1.3968, -0.9599] | 0.000 |
| Body size | 1413 | 1.714 | 1.713 | [1.5180, 1.9025] | 0.000 |
| Egg attendance (male) | 1725 | -0.042 | -0.041 | [-0.1735, 0.08726] | 0.265 |
| Egg attendance (female) | 2000 | 0.001 | 0.002 | [-0.1554, 0.1373] | 0.490 |
| Egg brooding | 2000 | -0.379 | -0.377 | [-0.6385, -0.1289] | 0.001 |
| Tadpole attendance (male) | 2000 | -0.262 | -0.259 | [-0.4965, -0.0245] | 0.013 |
| Tadpole attendance (female) | 1755 | 0.080 | 0.080 | [-0.3537, 0.4995] | 0.343 |
| Tadpole transport (male) | 2000 | -0.403 | -0.402 | [-0.7068, -0.1309] | 0.005 |
| Tadpole transport (female) | 2000 | -0.142 | -0.145 | [-0.4799, 0.1681] | 0.194 |
| Tadpole brooding | 2000 | 0.201 | 0.195 | [-0.1826, 0.6031] | 0.155 |
| Tadpole feeding | 2000 | -0.501 | -0.497 | [-0.7936, -0.1886] | 0.001 |
| Juvenile attendance | 2000 | -0.109 | -0.110 | [-0.3841, 0.1674] | 0.218 |
| Viviparity | 2000 | -0.149 | -0.172 | [-0.6896, 0.3572] | 0.248 |
| Terrestrial eggs | 1802 | -0.279 | -0.280 | [-0.3836, -0.1679] | 0.000 |
| Terrestrial larvae | 2000 | -0.548 | -0.542 | [-0.7834, -0.3326] | 0.000 |
| Direct development | 2000 | -0.323 | -0.323 | [-0.4603, -0.1742] | 0.001 |
| R^2^ | 1571 | 0.617 | 0.618 | [0.5679, 0.6596] | NA |
| λ | 1675 | 0.752 | 0.757 | [0.6394, 0.8694] | NA |
| Model likelihood | 1769 | -311.000 | -310.600 | [-336.0362, -288.1060] | NA |

| 1. **Clutch size: Reduced model** | | | | | |
| --- | --- | --- | --- | --- | --- |
| **Parameter** | **ESS** | **Mean** | **Median** | **95% HPD** | **P_x_** |
| Intercept | 1764 | -1.429 | -1.448 | [-1.9954, -0.9131] | 0.001 |
| Egg size | 1748 | -1.158 | -1.162 | [-1.3708, -0.9415] | 0.000 |
| Body size | 1469 | 1.713 | 1.714 | [1.5162, 1.8929] | 0.000 |
| Egg brooding | 2000 | -0.347 | -0.348 | [-0.6009, -0.1040] | 0.005 |
| Tadpole attendance (male) | 2000 | -0.308 | -0.311 | [-0.5383, -0.08152] | 0.005 |
| Tadpole transport (male) | 2000 | -0.410 | -0.418 | [-0.6670, -0.1369] | 0.006 |
| Tadpole feeding | 2000 | -0.540 | -0.538 | [-0.8351, -0.2521] | 0.001 |
| Terrestrial eggs | 1854 | -0.296 | -0.296 | [-0.3994, -0.1926] | 0.000 |
| Terrestrial larvae | 2000 | -0.485 | -0.486 | [-0.6796, -0.2878] | 0.000 |
| Direct development | 2000 | -0.339 | -0.337 | [-0.4830, -0.1886] | 0.000 |
| R^2^ | 1575 | 0.615 | 0.615 | [0.5720, 0.6593] | NA |
| λ | 1676 | 0.758 | 0.760 | [0.6350, 0.8615] | NA |
| Model likelihood | 1287 | -310.000 | -309.800 | [-331.8468, -287.8294] | NA |
