# Supplementary material for: Terrestrial reproduction and parental care drive rapid evolution in the trade-off between offspring size and number across amphibians
Source: PLoS Biol. 2022 Jan 4;20(1):e3001495. doi: 10.1371/journal.pbio.3001495 (PMC8726499; doi:10.1371/journal.pbio.3001495)
Supplement: S4 Table — Effect sizes are quantified as the median reduction in marginal likelihood when a given predictor is individually removed and the model rerun (see Methods, Identifying significant predictors of egg and clutch size evolution). The percentage change is computed as the difference between the estimated egg (A) or clutch size (B) when each predictor in turn is present compared to when it is absent, for an average-sized amphibian with average egg (A) or clutch size (B), holding all other predictors of the reduced models as absent. The direction of the percentage change reflects an increase (+) or decrease (−) in egg (A) or clutch size (B). (DOCX) [file pbio.3001495.s004.docx]

**S4 Table. Effect sizes of the significant variables in the reduced model for egg size (A) and clutch size (B) and corresponding percentage change in egg and clutch size for an average-sized amphibian.** Effect sizes are quantified as the median reduction in marginal likelihood when a given predictor is individually removed and the model re-run (see *Methods, Identifying significant predictors of egg and clutch size evolution*). The percentage change is computed as the difference between the estimated egg (A) or clutch size (B) when each predictor in turn is present compared to when it is absent, for an average sized amphibian with average egg (A) or clutch size (B), holding all other predictors of the reduced models as absent. The direction of the percentage change reflects an increase (+) or decrease (-) in egg (A) or clutch size (B).

| **(A) Egg size** | **Likelihood Reduction** | **% Change** |
| --- | --- | --- |
| Clutch size | 58.7 | NA |
| Body size | 74.8 | NA |
| Egg attendance (male) | 4.5 | +19.9 |
| Egg attendance (female) | 0.5 | +15.3 |
| Egg brooding | 6.6 | +57.4 |
| Tadpole attendance (female) | 4.3 | -49.6 |
| Tadpole feeding | 2.8 | -31.8 |
| Terrestrial eggs | 8.1 | +19.1 |
| Direct development | 10.9 | +39.0 |

| **(B) Clutch size** | **Likelihood Reduction** | | **% Change** |
| --- | --- | --- | --- |
| Egg size | 50.0 | NA | |
| Body size | 143.1 | NA | |
| Egg brooding | 6.1 | -55.0 | |
| Tadpole attendance (male) | 2.9 | -50.8 | |
| Tadpole transport (male) | 3.0 | -61.1 | |
| Tadpole feeding | 6.9 | -71.2 | |
| Terrestrial eggs | 17.1 | -49.4 | |
| Terrestrial larvae | 11.3 | -67.3 | |
| Direct development | 14.0 | -54.2 | |
