# Supplementary material for: Terrestrial reproduction and parental care drive rapid evolution in the trade-off between offspring size and number across amphibians
Source: PLoS Biol. 2022 Jan 4;20(1):e3001495. doi: 10.1371/journal.pbio.3001495 (PMC8726499; doi:10.1371/journal.pbio.3001495)
Supplement: S5 Table — We report the 19 branches that show exceptional rates of evolution in egg size, relative to the background rate, from the simple model including only body size and clutch size (left column) and the reduced model including the significant predictors (right column; the statistics for the reduced model is reported in full in S3B Table) (see Methods, Identifying rate shifts). Each branch is identified by its descendants. For each branch, we also report the median of the scalar r (see Methods, Identifying rate shifts). These branches correspond to those highlighted in red in Fig 2B and 2C. The blank cells listed under the reduced model are those that exhibit rate shifts in the simple model but not the reduced model, i.e., branches for which rapid egg size evolution can be attributed to the addition of parental care and reproductive ecology variables. The addition of terrestrial eggs, direct development, and parental care variables in the reduced model explains rapid egg size evolution in only 3 branches, that of Bufo valliceps, Eleutherodactylus alticola, and the clade consisting of Centrolene geckoideum, Centrolene savagei, Cochranella euknemos, Cochranella granulosa, Espadarana prosoblepon, Teratohyla midas, and Teratohyla spinosa. (DOCX) [file pbio.3001495.s005.docx]

S5 Table. Rate shifts in amphibian egg size evolution from variable rates model. We report the 19 branches that show exceptional rates of evolution in egg size, relative to the background rate, from the simple model including only body size and clutch size (left column) and the reduced model including the significant predictors (right column; the statistics for the reduced model is reported in full in S3B Table) (see *Methods, Identifying rate shifts*). Each branch is identified by its descendants. For each branch we also report the median of the scalar *r* (see *Methods, identifying rate shifts*). These branches correspond to those highlighted in red in Fig 2B and 2C. The blank cells listed under the reduced model are those that exhibit rate shifts in the simple model but not the reduced model, i.e. branches for which rapid egg size evolution can be attributed to the addition of parental care and reproductive ecology variables. The addition of terrestrial eggs, direct development and parental care variables in the reduced model explains rapid egg size evolution in only three branches, that of *Bufo valliceps*, *Eleutherodactylus alticola,* and the clade consisting of *Centrolene geckoideum*, *Centrolene savagei*, *Cochranella euknemos*, *Cochranella granulosa*, *Espadarana prosoblepon*, *Teratohyla midas*, and *Teratohyla spinosa.*

| Simple model | | Reduced model | |
| --- | --- | --- | --- |
| Descendant taxa | Median Rate (r) | Descendant taxa | Median Rate (r) |
| *Bufo valliceps* | 8.85 |  |  |
| *Leptodactylus ocellatus* | 11.11 | *Leptodactylus ocellatus* | 8.43 |
| *Centrolene geckoideum, Centrolene savagei, Cochranella euknemos, Cochranella granulosa, Espadarana prosoblepon, Teratohyla midas, Teratohyla spinosa* | 12.95 |  |  |
| *Cochranella euknemos, Cochranella granulosa, Espadarana prosoblepon, Teratohyla midas, Teratohyla spinosa* | 13.36 | *Cochranella euknemos, Cochranella granulosa, Espadarana prosoblepon, Teratohyla midas, Teratohyla spinosa* | 14.00 |
| *Cochranella euknemos, Cochranella granulosa, Espadarana prosoblepon* | 13.68 | *Cochranella euknemos, Cochranella granulosa, Espadarana prosoblepon* | 14.17 |
| *Cochranella euknemos, Cochranella granulosa* | 13.72 | *Cochranella euknemos, Cochranella granulosa* | 14.24 |
| *Cochranella granulosa* | 13.58 | *Cochranella granulosa* | 14.06 |
| *Cochranella euknemos* | 13.49 | *Cochranella euknemos* | 14.05 |
| *Espadarana prosoblepon* | 14.34 | *Espadarana prosoblepon* | 15.31 |
| *Teratohyla midas, Teratohyla spinosa* | 13.44 | *Teratohyla midas, Teratohyla spinosa* | 14.19 |
| *Teratohyla midas* | 13.43 | *Teratohyla midas* | 13.95 |
| *Teratohyla spinosa* | 13.70 | *Teratohyla spinosa* | 14.34 |
| *Centrolene geckoideum, Centrolene savagei* | 13.22 | *Centrolene geckoideum, Centrolene savagei* | 13.42 |
| *Centrolene geckoideum* | 12.91 | *Centrolene geckoideum* | 13.16 |
| *Centrolene savagei* | 13.31 | *Centrolene savagei* | 13.59 |
| *Eleutherodactylus alticola* | 11.05 |  |  |
| *Rana iberica* | 8.27 | *Rana iberica* | 7.80 |
| *Rana bergeri* | 14.80 | *Rana bergeri* | 13.89 |
| *Hyperolius frontalis* | 9.93 | *Hyperolius frontalis* | 9.66 |
|  |  | *Bufo fastidiosus* | 7.42 |
