# Supplementary material for: Terrestrial reproduction and parental care drive rapid evolution in the trade-off between offspring size and number across amphibians
Source: PLoS Biol. 2022 Jan 4;20(1):e3001495. doi: 10.1371/journal.pbio.3001495 (PMC8726499; doi:10.1371/journal.pbio.3001495)
Supplement: S6 Table — We report the 135 branches that show exceptional rates of evolution in clutch size, relative to the background rate, from the simple model including only body size and egg size (left column) and the reduced model also including the significant care and reproductive ecology predictors (right column; the statistics for the reduced model is reported in full in S3D Table). Each branch is identified by its descendants. For each branch, we also report the median of the scalar r (see Methods, Identifying rate shifts). These branches correspond to those highlighted in red in Fig 2E and 2F. The blank cells listed under the reduced model are those that exhibit rate shifts in the simple model but not the reduced model, i.e., branches for which rapid clutch size evolution can be attributed to the addition of parental care and reproductive ecology variables. The addition of direct development, offspring habitat, and parental care variables explains rapid clutch size evolution in 108 branches. (DOCX) [file pbio.3001495.s006.docx]

S6 Table. Rate shifts in amphibian clutch size evolution from variable rates model. We report the 135 branches that show exceptional rates of evolution in clutch size, relative to the background rate, from the simple model including only body size and egg size (left column) and the reduced model also including the significant care and reproductive ecology predictors (right column; the statistics for the reduced model is reported in full in S3D Table). Each branch is identified by its descendants. For each branch we also report the median of the scalar *r* (see *Methods, Identifying rate shifts*). These branches correspond to those highlighted in red in Fig 2E and 2F. The blank cells listed under the reduced model are those that exhibit rate shifts in the simple model but not the reduced model, i.e. branches for which rapid clutch size evolution can be attributed to the addition of parental care and reproductive ecology variables. The addition of direct development, offspring habitat and parental care variables explains rapid clutch size evolution in 108 branches.

| Simple model | | Reduced model | |
| --- | --- | --- | --- |
| Descendant taxa | Median Rate (*r*) | Descendant taxa | Median Rate (*r*) |
| *Pseudacris ornata* | 17.26 | *Pseudacris ornata* | 10.35 |
| *Aparasphenodon brunoi, Argenteohyla siemersi, Trachycephalus coriaceus, Trachycephalus mesophaeus, Trachycephalus venulosus* | 5.25 |  |  |
| *Trachycephalus coriaceus, Trachycephalus mesophaeus, Trachycephalus venulosus* | 4.53 |  |  |
| *Trachycephalus coriaceus, Trachycephalus mesophaeus* | 3.86 |  |  |
| *Trachycephalus mesophaeus* | 3.72 |  |  |
| *Trachycephalus coriaceus* | 3.74 |  |  |
| *Trachycephalus venulosus* | 3.86 |  |  |
| *Aparasphenodon brunoi, Argenteohyla siemersi* | 3.91 |  |  |
| *Aparasphenodon brunoi* | 3.80 |  |  |
| *Argenteohyla siemersi* | 5.33 |  |  |
| *Litoria ewingii, Litoria jervisiensis, Litoria verreauxii* | 12.74 |  |  |
| *Litoria ewingii, Litoria verreauxii* | 13.81 |  |  |
| *Litoria ewingii* | 12.81 |  |  |
| *Litoria verreauxii* | 14.26 |  |  |
| *Litoria jervisiensis* | 31.82 | *Litoria jervisiensis* | 19.53 |
| *Nectophrynoides minutus, Nectophrynoides tornieri, Nectophrynoides viviparus* | 6.48 |  |  |
| *Nectophrynoides minutus, Nectophrynoides tornieri* | 5.34 |  |  |
| *Nectophrynoides tornieri* | 6.12 |  |  |
| *Nectophrynoides minutus* | 4.68 |  |  |
| *Nectophrynoides viviparus* | 3.84 |  |  |
| *Bufo debilis, Bufo retiformis* | 5.21 |  |  |
| *Bufo debilis* | 4.82 |  |  |
| *Bufo retiformis* | 20.99 | *Bufo retiformis* | 11.31 |
| *Bufo valliceps* | 51.61 | *Bufo valliceps* | 31.54 |
| *Bufo marinus, Bufo schneideri* | 3.49 |  |  |
| *Bufo schneideri* | 5.64 |  |  |
| *Allobates femoralis, Allobates nidicola, Ameerega bilinguis, Ameerega hahneli, Ameerega parvula, Ameerega petersi, Ameerega picta, Ameerega silverstonei, Ameerega trivittata, Anomaloglossus beebei, Anomaloglossus stepheni, Dendrobates auratus, Dendrobates biolat, Dendrobates castaneoticus, Dendrobates histrionicus, Dendrobates lehmanni, Dendrobates leucomelas, Dendrobates pumilio, Dendrobates reticulatus, Dendrobates tinctorius, Dendrobates truncatus, Dendrobates ventrimaculatus, Epipedobates anthonyi, Epipedobates machalilla, Hyloxalus bocagei, Hyloxalus maculosus, Hyloxalus sauli, Hyloxalus subpunctatus, Hyloxalus toachi, Mannophryne trinitatis, Phyllobates terribilis, Phyllobates vittatus, Rheobates palmatus* | 16.32 |  |  |
| *Dendrobates biolat* | 5.16 |  |  |
| *Leptodactylus chaquensis, Leptodactylus ocellatus* | 4.14 |  |  |
| *Leptodactylus ocellatus* | 4.93 |  |  |
| *Leptodactylus chaquensis* | 14.28 |  |  |
| *Leptodactylus fallax* | 37.68 |  |  |
| *Odontophrynus americanus* | 8.07 |  |  |
| *Eleutherodactylus alticola* | 7.42 |  |  |
| *Rana bergeri, Rana iberica, Rana latastei, Rana macrocnemis, Rana perezi, Rana pyrenaica, Rana ridibunda, Rana saharica* | 7.73 |  |  |
| *Rana iberica, Rana latastei, Rana macrocnemis, Rana pyrenaica* | 8.84 | *Rana iberica, Rana latastei, Rana macrocnemis, Rana pyrenaica* | 4.63 |
| *Rana iberica, Rana macrocnemis, Rana pyrenaica* | 9.04 | *Rana iberica, Rana macrocnemis, Rana pyrenaica* | 4.76 |
| *Rana iberica, Rana pyrenaica* | 9.73 | *Rana iberica, Rana pyrenaica* | 5.09 |
| *Rana pyrenaica* | 9.26 | *Rana pyrenaica* | 4.87 |
| *Rana iberica* | 10.05 | *Rana iberica* | 5.09 |
| *Rana macrocnemis* | 8.80 | *Rana macrocnemis* | 4.63 |
| *Rana latastei* | 8.39 |  |  |
| *Rana bergeri, Rana perezi, Rana ridibunda, Rana saharica* | 10.03 | *Rana bergeri, Rana perezi, Rana ridibunda, Rana saharica* | 4.98 |
| *Rana perezi, Rana saharica* | 10.14 | *Rana perezi, Rana saharica* | 5.03 |
| *Rana saharica* | 10.28 | *Rana saharica* | 5.05 |
| *Rana perezi* | 10.06 | *Rana perezi* | 5.04 |
| *Rana bergeri, Rana ridibunda* | 11.11 | *Rana bergeri, Rana ridibunda* | 5.33 |
| *Rana ridibunda* | 10.60 | *Rana ridibunda* | 5.04 |
| *Rana bergeri* | 12.47 | *Rana bergeri* | 5.46 |
| *Rana areolata* | 3.08 |  |  |
| *Rana chiricahuensis* | 3.08 |  |  |
| *Rana narina, Rana supranarina, Rana swinhoana* | 3.34 |  |  |
| *Rana narina, Rana supranarina* | 3.36 |  |  |
| *Rana supranarina* | 3.28 |  |  |
| *Rana narina* | 3.28 |  |  |
| *Rana swinhoana* | 16.59 | *Rana swinhoana* | 10.91 |
| *Mantella laevigata* | 32.64 | *Mantella laevigata* | 8.85 |
| *Chaparana unculuanus, Euphlyctis cyanophlyctis, Euphlyctis hexadactylus, Fejervarya cancrivora, Fejervarya limnocharis, Hoplobatrachus occipitalis, Hoplobatrachus rugulosus, Hoplobatrachus tigerinus, Limnonectes blythii, Limnonectes finchi, Limnonectes kuhlii, Limnonectes palavanensis, Nanorana parkeri, Nanorana pleskei, Nanorana ventripunctata, Paa boulengeri, Paa liebigii, Paa shini, Paa spinosa, Paa yunnanensis, Sphaerotheca breviceps* | 3.04 |  |  |
| *Chaparana unculuanus, Limnonectes blythii, Limnonectes finchi, Limnonectes kuhlii, Limnonectes palavanensis, Nanorana parkeri, Nanorana pleskei, Nanorana ventripunctata, Paa boulengeri, Paa liebigii, Paa shini, Paa spinosa, Paa yunnanensis* | 3.27 |  |  |
| *Chaparana unculuanus, Nanorana parkeri, Nanorana pleskei, Nanorana ventripunctata, Paa boulengeri, Paa liebigii, Paa shini, Paa spinosa, Paa yunnanensis* | 3.52 |  |  |
| *Chaparana unculuanus, Nanorana parkeri, Nanorana pleskei, Nanorana ventripunctata, Paa liebigii, Paa yunnanensis* | 3.62 |  |  |
| *Chaparana unculuanus, Nanorana parkeri, Nanorana pleskei, Nanorana ventripunctata, Paa yunnanensis* | 3.83 |  |  |
| *Nanorana parkeri, Nanorana pleskei, Nanorana ventripunctata* | 3.74 |  |  |
| *Nanorana parkeri, Nanorana pleskei* | 3.81 |  |  |
| *Nanorana parkeri* | 3.74 |  |  |
| *Nanorana pleskei* | 3.77 |  |  |
| *Nanorana ventripunctata* | 3.73 |  |  |
| *Chaparana unculuanus, Paa yunnanensis* | 3.75 |  |  |
| *Paa yunnanensis* | 3.88 |  |  |
| *Chaparana unculuanus* | 3.68 |  |  |
| *Paa liebigii* | 4.27 | *Paa liebigii* | 3.14 |
| *Paa boulengeri, Paa shini, Paa spinosa* | 4.09 |  |  |
| *Paa boulengeri, Paa shini* | 5.47 | *Paa boulengeri, Paa shini* | 3.74 |
| *Paa boulengeri* | 5.27 | *Paa boulengeri* | 3.60 |
| *Paa shini* | 11.96 | *Paa shini* | 9.66 |
| *Paa spinosa* | 3.98 |  |  |
| *Limnonectes blythii, Limnonectes finchi, Limnonectes kuhlii, Limnonectes palavanensis* | 3.34 |  |  |
| *Limnonectes blythii, Limnonectes finchi, Limnonectes palavanensis* | 3.40 |  |  |
| *Limnonectes blythii, Limnonectes finchi* | 3.52 |  |  |
| *Limnonectes blythii* | 3.67 |  |  |
| *Limnonectes finchi* | 3.53 |  |  |
| *Limnonectes palavanensis* | 3.38 |  |  |
| *Limnonectes kuhlii* | 3.36 |  |  |
| *Euphlyctis cyanophlyctis, Euphlyctis hexadactylus, Fejervarya cancrivora, Fejervarya limnocharis, Hoplobatrachus occipitalis, Hoplobatrachus rugulosus, Hoplobatrachus tigerinus, Sphaerotheca breviceps* | 3.08 |  |  |
| *Euphlyctis cyanophlyctis, Euphlyctis hexadactylus, Hoplobatrachus occipitalis, Hoplobatrachus rugulosus, Hoplobatrachus tigerinus* | 3.15 |  |  |
| *Hoplobatrachus occipitalis, Hoplobatrachus rugulosus, Hoplobatrachus tigerinus* | 3.73 |  |  |
| *Hoplobatrachus rugulosus, Hoplobatrachus tigerinus* | 3.34 |  |  |
| *Hoplobatrachus rugulosus* | 3.22 |  |  |
| *Hoplobatrachus tigerinus* | 3.21 |  |  |
| *Hoplobatrachus occipitalis* | 3.25 |  |  |
| *Euphlyctis cyanophlyctis, Euphlyctis hexadactylus* | 3.23 |  |  |
| *Euphlyctis hexadactylus* | 3.15 |  |  |
| *Euphlyctis cyanophlyctis* | 3.15 |  |  |
| *Fejervarya cancrivora, Fejervarya limnocharis, Sphaerotheca breviceps* | 3.28 |  |  |
| *Fejervarya cancrivora, Fejervarya limnocharis* | 3.35 |  |  |
| *Fejervarya cancrivora* | 3.36 |  |  |
| *Fejervarya limnocharis* | 3.34 |  |  |
| *Sphaerotheca breviceps* | 3.53 |  |  |
| *Tomopterna krugerensis, Tomopterna marmorata* | 3.58 |  |  |
| *Tomopterna krugerensis* | 4.94 |  |  |
| *Tomopterna marmorata* | 3.76 |  |  |
| *Conraua goliath* | 6.06 | *Conraua goliath* | 12.59 |
| *Austrochaperina palmipes, Callulops pullifer, Callulops robustus, Hylophorbus rufescens, Kalophrynus pleurostigma, Kaloula pulchra, Microhyla heymonsi, Microhyla ornata, Microhyla pulchra, Synapturanus mirandaribeiroi* | 3.89 |  |  |
| *Austrochaperina palmipes, Callulops pullifer, Callulops robustus, Hylophorbus rufescens, Kalophrynus pleurostigma, Kaloula pulchra, Microhyla heymonsi, Microhyla ornata, Microhyla pulchra* | 5.18 |  |  |
| *Austrochaperina palmipes, Callulops pullifer, Callulops robustus, Hylophorbus rufescens, Kalophrynus pleurostigma* | 6.98 |  |  |
| *Austrochaperina palmipes, Callulops pullifer, Callulops robustus, Hylophorbus rufescens* | 8.04 |  |  |
| *Austrochaperina palmipes, Callulops robustus, Hylophorbus rufescens* | 7.29 |  |  |
| *Austrochaperina palmipes, Hylophorbus rufescens* | 7.30 |  |  |
| *Hylophorbus rufescens* | 7.08 |  |  |
| *Austrochaperina palmipes* | 7.24 |  |  |
| *Callulops robustus* | 7.10 |  |  |
| *Callulops pullifer* | 7.21 |  |  |
| *Kalophrynus pleurostigma* | 8.43 |  |  |
| *Kaloula pulchra, Microhyla heymonsi, Microhyla ornata, Microhyla pulchra* | 5.32 |  |  |
| *Microhyla heymonsi, Microhyla ornata, Microhyla pulchra* | 5.18 |  |  |
| *Microhyla heymonsi, Microhyla ornata* | 5.52 |  |  |
| *Microhyla heymonsi* | 5.33 |  |  |
| *Microhyla ornata* | 5.31 |  |  |
| *Microhyla pulchra* | 5.43 |  |  |
| *Kaloula pulchra* | 5.28 |  |  |
| *Synapturanus mirandaribeiroi* | 5.81 |  |  |
| *Laotriton laoensis, Pachytriton brevipes, Pachytriton labiatus* | 5.25 |  |  |
| *Pachytriton brevipes, Pachytriton labiatus* | 5.14 |  |  |
| *Pachytriton labiatus* | 5.08 |  |  |
| *Pachytriton brevipes* | 5.13 |  |  |
| *Laotriton laoensis* | 6.51 | *Laotriton laoensis* | 5.39 |
| *Taricha rivularis* | 5.19 |  |  |
| *Chioglossa lusitanica, Mertensiella caucasica, Salamandra atra, Salamandra salamandra* | 5.13 |  |  |
| *Chioglossa lusitanica, Mertensiella caucasica* | 5.32 |  |  |
| *Mertensiella caucasica* | 5.93 | *Mertensiella caucasica* | 9.33 |
| *Chioglossa lusitanica* | 5.28 |  |  |
| *Salamandra atra, Salamandra salamandra* | 5.37 |  |  |
| *Salamandra salamandra* | 5.17 |  |  |
| *Salamandra atra* | 5.47 |  |  |
| *Ambystoma mabeei* | 38.75 | *Ambystoma mabeei* | 19.38 |
|  |  | *Anura* | 19.96 |
|  |  | *Phyllodytes luteolus* | 5.14 |
