# Supplementary material for: Terrestrial reproduction and parental care drive rapid evolution in the trade-off between offspring size and number across amphibians
Source: PLoS Biol. 2022 Jan 4;20(1):e3001495. doi: 10.1371/journal.pbio.3001495 (PMC8726499; doi:10.1371/journal.pbio.3001495)
Supplement: S7 Table — VIF, variance inflation factor. (DOCX) [file pbio.3001495.s007.docx]

**S7 Table. Variance inflation factors (VIF) for the full models for egg size (A) and clutch size (B).**

| **(A) Egg size: full model** |  |
| --- | --- |
| **Predictor** | **VIF** |
| Body size | 1.435 |
| Clutch size | 2.395 |
| Egg attendance (male) | 1.946 |
| Egg attendance (female) | 1.993 |
| Egg brooding | 1.155 |
| Tadpole attendance (male) | 1.212 |
| Tadpole attendance (female) | 1.250 |
| Tadpole transport (male) | 1.761 |
| Tadpole transport (female) | 1.233 |
| Tadpole brooding | 1.460 |
| Tadpole feeding | 1.200 |
| Juvenile attendance | 1.123 |
| Viviparity | 1.123 |
| Terrestrial eggs | 2.195 |
| Terrestrial larvae | 1.636 |
| Direct development | 1.953 |

| **(B) Clutch size: full model** |  |
| --- | --- |
| **Predictor** | **VIF** |
| Body size | 1.615 |
| Egg size | 2.367 |
| Egg attendance (male) | 2.020 |
| Egg attendance (female) | 1.800 |
| Egg brooding | 1.182 |
| Tadpole attendance (male) | 1.202 |
| Tadpole attendance (female) | 1.240 |
| Tadpole transport (male) | 1.729 |
| Tadpole transport (female) | 1.231 |
| Tadpole brooding | 1.471 |
| Tadpole feeding | 1.217 |
| Juvenile attendance | 1.121 |
| Viviparity | 1.128 |
| Terrestrial eggs | 2.179 |
| Terrestrial larvae | 1.630 |
| Direct development | 2.050 |
