# Supplementary material for: Terrestrial reproduction and parental care drive rapid evolution in the trade-off between offspring size and number across amphibians
Source: PLoS Biol. 2022 Jan 4;20(1):e3001495. doi: 10.1371/journal.pbio.3001495 (PMC8726499; doi:10.1371/journal.pbio.3001495)
Supplement: S3 Data — (DOCX) [file pbio.3001495.s010.docx]

**S3 Data. Supplementary Data Reference List.**

1 Abrunhosa, P. A. & Wogel, H. Breeding behavior of the leaf-frog Phyllomedusa burmeisteri (Anura: Hylidae). Amphibia-Reptilia 25, 125-135 (2004).

2 Acioli, E. C. S. & Neckel-Oliveira, S. Reproductive biology of Ameerega trivittata (Anura: Dendrobatidae) in an area of terra firme forest in eastern Amazonia. Acta Amazonica 44, 473-480 (2014).

3 Aichinger, M. Tadpole transport in relation to rainfall, fecundity and body size in five species of poison-dart frogs from Amazonian Peru. Amphibia-Reptilia 12, 49-55 (1991).

4 Akiyama, S., Iwao, Y. & Miura, I. Evidence for true fall-mating in Japanese newt Cynops pyrrhogaster. Zoological Science 28, 758-763 (2011).

5 Alcala, A. C. Breeding behavior and early development of frogs of Negros, Philippine Islands. Copeia 1962, 679-726 (1962).

6 Alcher, M. Reproduction en élevage de Euproctus platycephalus (Urodela, Salamandridae). Amphibia-Reptilia 2, 97-105 (1981).

7 Alfimov, A. V. & Berman, D. I. Reproduction of the Siberian salamander, Salamandrella keyserlingii (Amphibia, Caudata, Hynobiidae), in water bodies on permafrost in Northeastern Asia. Biology Bulletin 37, 807-822 (2010).

8 Allen, W. L., Street, S. E. & Capellini, I. Fast life history traits promote invasion success in amphibians and reptiles. Ecology Letters 20, 222-230 (2017).

9 Altig, R. Notes on the breeding biology of four species of mantellid frogs from Madagascar. Tropical Zoology 21, 187-194 (2008).

10 Altig, R. & Rowley, J. J. L. The breeding behavior of Glyphoglossus molossus and the tadpoles of Glyphoglossus molossus and Calluella guttulata (Microhylidae). Zootaxa 3811, 381-386 (2014).

11 Amburgey, S., Funk, W. C., Murphy, M. & Muths, E. Effects of hydroperiod duration on survival, developmental rate, and size at metamorphosis in boreal chorus frog tadpoles (Pseudacris maculata). Herpetologica 68, 456-467 (2012).

12 AmphibiaWeb. <https://amphibiaweb.org>, 2018).

13 Anderson, A. S., Monasterio, C. & Schoo, L. Breeding behaviour of the poorly known Australian hylid frog Litoria longirostris. Herpetofauna 40, 9-12 (2010).

14 Anderson, J. D. & Williamson, G. K. Terrestrial mode of reproduction in Ambystoma cingulatum. Herpetologica 32, 214-221 (1976).

15 Andreone, F., Vences, M., Guarino, F. M., Glaw, F. & Randrianirina, J. E. Natural history and larval morphology of Boophis occidentalis (Anura: Mantellidae: Boophinae) provide new insights into the phylogeny and adaptive radiation of endemic Malagasy frogs. Journal of Zoology 257, 425-438 (2002).

16 Anstis, M. Direct development in the Australian myobatrachid frog Metacrinia nichollsi from Western Australia. Records of the Western Australian Museum 24, 133-150 (2008).

17 Ao, M. & Bordoloi, S. Annual breeding cycle and spawning behaviour of Hyla annectans Jerdon 1870 in Nagaland, India. Current Science 79, 943-945 (2000).

18 Arikan, H., Tosunoğlu, M., Atatür, M. K. & Göçmen, B. Some Comments on the Breeding Biology of Pelodytes caucasicus Boulenger, 1896 (Anura: Pelodytidae) from Uzungöl, Northeast Anatolia. Turkish Journal of Zoology 31, 53-64 (2007).

19 Ashton, R. E. & Braswell, A. L. Nest and larvae of the Neuse River waterdog, Necturus lewisi (Brimley)(Amphibia: Proteidae). Brimleyana 1, 15-22 (1979).

20 Babbitt, K. J. & Meshaka Jr, W. E. Benefits of eating conspecifics: effects of background diet on survival and metamorphosis in the Cuban treefrog (Osteopilus septentrionalis). Copeia 2000, 469-474 (2000).

21 Bahir, M. M., Meegaskumbura, M., Manamendra-Arachchi, K., Schneider, C. J. & Pethiyagoda, R. Reproduction and terrestrial direct development in Sri Lankan shrub frogs (Ranidae: Rhacophorinae: Philautus). The Raffles Bulletin of Zoology Supplement No. 12, 339-350 (2005).

22 Barroso, D. D. & Bogaerts, S. in Herpetologia Candiana (eds P Lymberakis, E Valakos, P Pafilis, & M Mylonas) 147-151 (S.E.H., 2001).

23 Başkale, E., Sayım, F. & Kaya, U. Body size and reproductive characteristics of paedomorphic and metamorphic individuals of the northern banded newt (Ommatotriton ophryticus). Acta Herpetologica 6, 19-25 (2011).

24 Bei, Y. et al. First record of nest site and egg guarding in the caecilian Ichthyophis bannanicus (Amphibia: Gymnophiona: Ichthyophiidae). Journal of Natural History 46, 859-865 (2012).

25 Bell, B. D. & Wassersug, R. J. Anatomical features of Leiopelma embryos and larvae: implications for anuran evolution. Journal of Morphology 256, 160-170 (2003).

26 Bickford, D. Animal behaviour: male parenting of New Guinea froglets. Nature 418, 601-602 (2002).

27 Bickford, D. P. Differential parental care behaviors of arboreal and terrestrial microhylid frogs from Papua New Guinea. Behavioral Ecology and Sociobiology 55, 402-409 (2004).

28 Biju, S. D. Reproductive mode in the shrub frog Philautus glandulosus (Jerdon, 1853)(Anura: Rhacophoridae). Current Science 84, 283-284 (2003).

29 Biju, S. D. A novel nesting behaviour of a treefrog, Rhacophorus lateralis in the Western Ghats, India. Current Science 97, 433-437 (2009).

30 Biju, S. D. et al. Frankixalus, a new Rhacophorid genus of tree hole breeding frogs with oophagous tadpoles. PLoS One 11, e0145727 (2016).

31 Biju, S. D. et al. A taxonomic review of the Night Frog genus Nyctibatrachus Boulenger, 1882 in the Western Ghats, India (Anura: Nyctibatrachidae) with description of twelve new species. Zootaxa 3029, 1-96 (2011).

32 Bille, T. Eggs and hatchlings of the Mexican salamander Pseudoeurycea cephalica (Caudata: Plethodontidae). Revista de Biología Tropical 46, 447-452 (1998).

33 Bitar, Y. O., Pinheiro, L. P., Abe, P. S. & Santos-Costa, M. C. Santos-Costa. Species composition and reproductive modes of anurans from a transitional Amazonian forest, Brazil. Zoologia 29, 19-26 (2012).

34 Blackburn, D. C., Brecko, J., Stanley, E. L. & Meirte, D. Observations on the reproductive biology of Laurentophryne parkeri (Laurent, 1950) based on the holotype. Herpetology Notes 10, 681-683 (2017).

35 Blommers-Schlösser, R. M. A. Observations on the larval development of some Malagasy frogs, with notes on their ecology and biology (Anura: Dyscophinae, Scaphiophryninae and Cophylinae). Beaufortia 24, 7-26 (1975).

36 Blommers-Schlösser, R. M. A. A unique case of mating behaviour in a Malagasy tree frog, Gephyromantis liber (Peracca, 1893), with observations on the larval development (Amphibia, Ranidae). Beaufortia 23, 15-25 (1975).

37 Bosch, R. A., García, L. Y., Domínguez, S. D. C. & Martínez, E. L. T. Clutches, nest attendance, and hatching in a rock-cave-dwelling frog, Eleutherodactylus (Syrrophus) zeus, from Cuba. Journal of Cave and Karst Studies 77, 83-86 (2015).

38 Boscherini, A. & Romano, A. Parental care in Salamandrina perspicillata (Amphibia, Salamandridae): egg defence against caddisfly larvae. North-Western Journal of Zoology 7, 167-170 (2011).

39 Boulenger, G. A. 2. Description of a new Tree‐Frog of the Genus Hyla, from British Guiana, carrying eggs on the back. Proceedings of the Zoological Society of London 74, 106 (1904).

40 Bourne, G. R. Reproductive behavior of terrestrial breeding frogs Eleutherodactylus johnstonei in Guyana. Journal of Herpetology 31, 221-229 (1997).

41 Bourne, G. R., Collins, A. C., Holder, A. M. & McCarthy, C. L. Vocal communication and reproductive behavior of the frog Colostethus beebei in Guyana. Journal of Herpetology 35, 272-281 (2001).

42 Brasileiro, C. A. & Martins, M. Breeding biology of Physalaemus centralis Bokermann, (Anura: Leptodactylidae) in southeastern Brazil. Journal of Natural History 40, 1199-1209 (2006).

43 Briggler, J. T. & Puckette, W. L. Observations on reproductive biology and brooding behavior of the Ozark zigzag salamander, Plethodon angusticlavius. The Southwestern Naturalist 48, 96-100 (2003).

44 Briggs, V. S. Mating patterns of red‐eyed treefrogs, Agalychnis callidryas and A. moreletii. Ethology 114, 489-498 (2008).

45 Briggs, V. S. Do big dads make big babies? Paternal effects on larval performance in red-eyed treefrogs of Belize (Agalychnis callidryas, A. moreletti). The Herpetological Journal 23, 131-138 (2013).

46 Brown, J. L., Morales, V. & Summers, K. Divergence in parental care, habitat selection and larval life history between two species of Peruvian poison frogs: an experimental analysis. Journal of Evolutionary Biology 21, 1534-1543 (2008).

47 Brown, J. L., Morales, V. & Summers, K. A key ecological trait drove the evolution of biparental care and monogamy in an amphibian. The American Naturalist 175, 436-446 (2010).

48 Brown, J. L. et al. A taxonomic revision of the Neotropical poison frog genus Ranitomeya (Amphibia: Dendrobatidae). Zootaxa 3083, 1-120 (2011).

49 Brown, J. L., Twomey, E., Morales, V. & Summers, K. Phytotelm size in relation to parental care and mating strategies in two species of Peruvian poison frogs. Behaviour 145, 1139-1165 (2008).

50 Brown, J. L., Twomey, E., Pepper, M. & Rodriguez, M. S. Revision of the Ranitomeya fantastica species complex with description of two new species from Central Peru (Anura: Dendrobatidae). Zootaxa 1823, 1-24 (2008).

51 Brown, R. M. & Iskandar, D. T. Nest site selection, larval hatching, and advertisement calls, of Rana arathooni from southwestern Sulawesi (Celebes) Island, Indonesia. Journal of Herpetology 34, 404-413 (2000).

52 Browne, R. K. et al. The giant salamanders (Cryptobranchidae): Part B. Biogeography, ecology and reproduction. Amphibian and Reptile Conservation 5, 30-50 (2014).

53 Brunetti, A. E., Taboada, C. A. & Faivovich, J. The reproductive biology of Hypsiboas punctatus (Anura: Hylidae): male territoriality and the possible role of different signals during female choice. Salamandra 50, 215-224 (2014).

54 Brust, D. G. Maternal brood care by Dendrobates pumilio: a frog that feeds its young. Journal of Herpetology 27, 96-98 (1993).

55 Buckley, D. in eLS (John Wiley & Sons, Ltd, 2012).

56 Buckley, D., Alcobendas, M. & García-París, M. in Evolución y Adaptación 150 años después del Origen de las Especies (Sociedad Española de Biología Evolutiva, 2009).

57 Buckley, D., Alcobendas, M., García‐París, M. & Wake, M. H. Heterochrony, cannibalism, and the evolution of viviparity in Salamandra salamandra. Evolution & Development 9, 105-115 (2007).

58 Cabanzo-Olarte, L. C., Ramírez-Pinilla, M. P. & Serrano-Cardozo, V. H. Oviposition, site preference, and evaluation of male clutch attendance in Espadarana andina (Anura: Centrolenidae). Journal of Herpetology 47, 314-320 (2013).

59 Cajade, R., Schaefer, E. F., Duré, M. I., Kehr, A. I. & Marangoni, F. Reproductive biology of Argenteohyla siemersi pederseni Williams and Bosso,(Anura: Hylidae) in northeastern Argentina. Journal of Natural History 44, 1953-1978 (2010).

60 Caldwell, J. P. Pair bonding in spotted poison frogs. Nature 385, 211 (1997).

61 Caldwell, J. P. & de Araujo, M. C. Cannibalistic Interactions Resulting from Indiscriminate Predatory Behavior in Tadpoles of Poison Frogs (Anura: Dendrobatidae). Biotropica 30, 92-103 (1998).

62 Caldwell, J. P. & de Oliveira, V. R. L. Determinants of biparental care in the spotted poison frog, Dendrobates vanzolinii (Anura: Dendrobatidae). Copeia 1999, 565-575 (1999).

63 Caldwell, J. P. & Lima, A. P. A new Amazonian species of Colostethus (Anura: Dendrobatidae) with a nidicolous tadpole. Herpetologica 59, 219-234 (2003).

64 Canterbury, R. A. & Pauley, T. K. Time of mating and egg deposition of West Virginia populations of the salamander Aneides aeneus. Journal of Herpetology 28, 431-434 (1994).

65 Carvalho Jr, R. R., Galdino, C. A. B. & Nascimento, L. B. Notes on the courtship behavior of Aplastodiscus arildae (Cruz & Peixoto, 1985) at an urban forest fragment in southeastern Brazil (Amphibia, Anura, Hylidae). Arquivos do Museu Nacional, Rio de Janeiro 64, 247-254 (2006).

66 Cassiano-Lima, D., Borges-Nojosa, D. M., Cascon, P. & Cechin, S. Z. The reproductive mode of Adelophryne maranguapensis Hoogmoed, Borges & Cascon, 1994, (Anura, Eleutherodactylidae) an endemic and threatened species from Atlantic Forest remnants in northern Brazil. North-Western Journal of Zoology 7, 92-97 (2011).

67 Castillo‐Trenn, P. & Coloma, L. A. Notes on behaviour and reproduction in captive Allobates kingsburyi (Anura: Dendrobatidae), with comments on evolution of reproductive amplexus. International Zoo Yearbook 42, 58-70 (2008).

68 Channing, A. Observations on the natural history of Stephopaedes anotis (Bufonidae). Journal of Herpetology 27, 213-214 (1993).

69 Channing, A., Menegon, M., Salvidio, S. & Akker, S. A new forest toad from the Ukaguru Mountains, Tanzania (Bufonidae: Nectophrynoides). African Journal of Herpetology 54, 149-157 (2005).

70 Charney, N. D., Castorino, J. J., Dobro, M. J. & Steely, S. L. Embryo development inside female salamander (Ambystoma jeffersonianum-laterale) prior to egg laying. PloS One 9, e91919 (2014).

71 Chen, C. et al. The breeding ecology of a critically endangered salamander, Hynobius amjiensis (Caudata: Hynobiidae), endemic to eastern China. Asian Herpetological Research 7, 53-58 (2016).

72 Coloma, L. A. in Miscellaneous Publication No. 87 1-72 (Natural History Museum, University of Kansas, 1995).

73 Consolmagno, R. C., Requena, G. S., Machado, G. & Brasileiro, C. A. Costs and benefits of temporary egg desertion in a rocky shore frog with male-only care. Behavioral Ecology and Sociobiology 70, 785-795 (2016).

74 Cook, C. L., Ferguson, J. W. H. & Telford, S. R. Adaptive male parental care in the giant bullfrog, Pyxicephalus adspersus. Journal of Herpetology 35, 310-315 (2001).

75 Crump, M. L. Possible enhancement of growth in tadpoles through cannibalism. Copeia 1990, 560-564 (1990).

76 Crump, M. L. in Amphibian biology Vol. 2 (ed Harold Heatwole) 518-567 (Surrey Beatty & Sons, 1995).

77 Crump, M. L. in Parental Care: Evolution, Mechanisms, and Adaptive Significance Vol. 25 (eds Jay S Rosenblatt & Charles T Snowdon) 109-144 (Academic Press, 1996).

78 Crump, M. L. Anuran reproductive modes: evolving perspectives. Journal of Herpetology 49, 1-16 (2015).

79 Dalgetty, L. & Kennedy, M. W. Building a home from foam—túngara frog foam nest architecture and three-phase construction process. Biology Letters 6, 293-296 (2010).

80 Das, I. & Deuti, K. Notes on date of publication and generic identity of Leptobrachium nokrekensis Mathew and Sen,“2009” 2010 (Amphibia: Anura: Megophryidae). Current Herpetology 30, 69-73 (2011).

81 Davenport, J. M. & Summers, K. Environmental influences on egg and clutch sizes in lentic-and lotic-breeding salamanders. Phyllomedusa 9, 87-98 (2010).

82 De La Riva, I. & Burrowes, P. A. A new species of Psychrophrynella (Anura: Craugastoridae) from the Cordillera Real, Department La Paz, Bolivia. Zootaxa 3887, 459-470 (2014).

83 De Lima, A. V. P. et al. Developmental aspects of the direct‐developing frog Adelophryne maranguapensis. genesis 54, 257-271 (2016).

84 De Oliveira, F. F. R. Mating behaviour, territoriality and natural history notes of Phyllomedusa ayeaye Lutz, 1966 (Hylidae: Phyllomedusinae) in south-eastern Brazil. Journal of Natural History 51, 657-675 (2017).

85 De Oliveira, F. F. R., Nogueira, P. A. G. & Eterovick, P. C. Natural history of Phyllomedusa megacephala (Miranda-Ribeiro, 1926)(Anura: Hylidae) in southeastern Brazil, with descriptions of its breeding biology and male territorial behaviour. Journal of Natural History 46, 117-129 (2012).

86 De Oliveira Filho, J. C. & Giaretta, A. A. Reproductive behavior of Leptodactylus mystacinus (Anura, Leptodactylidae) with notes on courtship call of other Leptodactylus species. Iheringia. Série Zoologia 98, 508-515 (2008).

87 De Sa, R. O., Brandão, R. & Guimarães, L. D. a. Description of the tadpole of Leptodactylus pustulatus Peters, 1870 (Anura: Leptodactylidae). Zootaxa 1523, 49-58 (2007).

88 De Sá, R. O. et al. Systematics of the neotropical genus Leptodactylus Fitzinger, 1826 (Anura: Leptodactylidae): Phylogeny, the relevance of non-molecular evidence, and species accounts. South American Journal of Herpetology 9, S1-S128, doi:10.2994/SAJH-D-13-00022.1 (2014).

89 de Sá, R. O., Loader, S. P. & Channing, A. A new species of Callulina (Anura: Microhylidae) from the West Usambara Mountains, Tanzania. Journal of Herpetology 38, 219-224 (2004).

90 del Pino, E. M. Morphology of the pouch and incubatory integument in marsupial frogs (Hylidae). Copeia 1, 10-17 (1980).

91 Delia, J., Bravo‐Valencia, L. & Warkentin, K. M. Patterns of parental care in Neotropical glassfrogs: fieldwork alters hypotheses of sex‐role evolution. Journal of Evolutionary Biology 30, 898-914 (2017).

92 Della Rocca, F., Vignoli, L. & Bologna, M. A. The reproductive biology of Salamandrina terdigitata (Caudata, Salamandridae). The Herpetological Journal 15, 273-278 (2005).

93 Dias, P. H. d. S., Hepp, F. S. F. d. S., Silva, A. M. P. T. d. C. e. & Silva, S. P. d. C. e. Breeding biology and advertisement call of the horned leaf-frog, Proceratophrys appendiculata (Amphibia: Anura: Odontophrynidae). Zoologia (Curitiba) 30, 388-396 (2013).

94 Dias, T. M. et al. Breeding biology, territoriality, and reproductive site use by Phyllomedusa iheringii (Anura: Phyllomedusidae) from the South American Pampa in Brazil. Salamandra 53, 257-266 (2017).

95 Dias, T. M., Maragno, F. P., Madalozz, B., Prado, C. & Cechin, S. Z. Breeding sites of the leaf frog Phyllomedusa tetraploidea (Hylidae, Phyllomedusinae) in a forest remnant in southern Brazil. North-Western Journal of Zoology 9, 422-424 (2013).

96 Diaz, N. F., Sallaberry, M. & Valencia, J. Microhabitat and reproductive traits in populations of the frog, Batrachyla taeniata. Journal of Herpetology 21, 317-323 (1987).

97 Díaz-Paniagua, C. Oviposition behavior of Triturus marmoratus pygmaeus. Journal of Herpetology 23, 159-163 (1989).

98 Diesel, R., Bäurle, G. & Vogel, P. Cave breeding and froglet transport: a novel pattern of anuran brood care in the Jamaican frog, Eleutherodactylus cundalli. Copeia 1995, 354-360 (1995).

99 Domenico, E. A., Haddad, C. F. & Zaher, H. Natural history of Paratelmatobius gaigeae (Amphibia, Anura, Leptodactylidae): description of the tadpole and advertisement call. Journal of Herpetology 48, 430-433 (2014).

100 Donnelly, M. A. & Wake, M. H. A new Microcaecilia (Amphibia: Gymnophiona) from Guyana with comments on Epicrionops niger. Copeia 2013, 223-231 (2013).

101 Dopazo, H. & Alberch, P. Preliminary results on optional viviparity and intrauterine siblicide in Salamandra salamandra populations from Northern Spain. Mertensiella 4, 125-137 (1994).

102 Duellman, W. E. The hylid frogs of Middle America. Monograph of the Museum of Natural History, The University of Kansas 2, 1-753 (1970).

103 Duellman, W. E. Taxonomy of Brazilian hylid frogs of the genus Gastrotheca. Journal of Herpetology 18, 302-312 (1984).

104 Duellman, W. E. Two new species of marsupial frogs (Anura: Hylidae) from Peru. Copeia 1987, 903-909 (1987).

105 Duellman, W. E., Cadle, J. E. & Cannatella, D. C. A new species of terrestrial Phyllomedusa (Anura: Hylidae) from southern Peru. Herpetologica 44, 91-95 (1988).

106 Duellman, W. E. & Chavéz, G. Reproduction in the marsupial frog Gastrotheca testudinea (Anura: Hemiphractidae). Herpetology Notes 3, 87-90 (2010).

107 Duellman, W. E. & Gray, P. Developmental biology and systematics of the egg-brooding hylid frogs, genera Flectonotus and Fritziana. Herpetologica 39, 333-359 (1983).

108 Duellman, W. E. & Hoogmoed, M. S. The taxonomy and phylogenetic relationships of the hylid frog genus Stefania. Miscellaneous Publications The University of Kansas Museum of Natural History 75, 1-39 (1984).

109 Duellman, W. E. & Maness, S. J. The reproductive behavior of some hylid marsupial frogs. Journal of Herpetology 14, 213-222 (1980).

110 Duellman, W. E. & Trueb, L. Biology of Amphibians. 670 (McGraw-Hill, 1986).

111 Duellman, W. E. & Trueb, L. Cryptic species of hylid marsupial frogs in Peru. Journal of Herpetology 22, 159-179 (1988).

112 Dugas, M. B., Wamelink, C. N., Killius, A. M. & Richards-Zawacki, C. L. Parental care is beneficial for offspring, costly for mothers, and limited by family size in an egg-feeding frog. Behavioral Ecology 27, 476-483 (2016).

113 Dulac, C., O’Connell, L. A. & Wu, Z. Neural control of maternal and paternal behaviors. Science 345, 765-770 (2014).

114 Dunn, E. R. The amphibian and reptilian fauna of bromeliads in Costa Rica and Panama. Copeia 1937, 163-167 (1937).

115 Emerson, S. B. A macroevolutionary study of historical contingency in the fanged frogs of Southeast Asia. Biological Journal of the Linnean Society 73, 139-151 (2001).

116 Engelbrecht, D., Mashao, M. & Halajian, A. Notes on the breeding behaviour and ecology of edible bullfrogs Pyxicephalus edulis Peters, 1854 in the Limpopo Province, South Africa. Herpetology Notes 8, 365-369 (2015).

117 Exbrayat, J.-M. in Reproductive Biology and Phylogeny of Gymnophiona (Caecilians) (ed Jean-Marie Exbrayat) 303-324 (Science Publishers, 2006).

118 Ferraro, D. P., Pereyra, M. E., Baldo, J. D. & Faivovich, J. The clutch structure of Pleurodema tucumanum (Anura: Leptodactylidae). Salamandra 52, 48-52 (2016).

119 Formas, J. R. & Pugin, E. Tadpoles of Eupsophus roseus and Bufo variegatus (Amphibia, Anura) in southern Chile. Journal of Herpetology 12, 243-246 (1978).

120 Formas, J. R. & Vera, M. A. Reproductive patterns of Eupsophus roseus and E. vittatus. Journal of Herpetology 14, 11-14 (1980).

121 Forti, L. R., Mott, T. & Strüssmann, C. Breeding biology of Ameerega braccata (Steindachner, 1864)(Anura: Dendrobatidae) in the Cerrado of Brazil. Journal of Natural History 47, 2363-2371 (2013).

122 Fukuyama, K. & Kusano, T. Factors affecting breeding activity in a stream-breeding frog, Buergeria buergeri. Journal of Herpetology 26, 88-91 (1992).

123 Funk, W. C., Fletcher-Lazo, G., Nogales-Sornosa, F. & Almeida-Reinoso, D. First description of a clutch and nest site for the genus Caecilia (Gymnophiona: Caeciliidae). Herpetological Review 35, 128-130 (2004).

124 Gascon, C. Breeding of Leptodactylus knudseni: responses to rainfall variation. Copeia 1991, 248-252 (1991).

125 Giaretta, A. A. Reproductive specializations of the bromeliad hylid frog Phyllodytes luteolus. Journal of Herpetology 30, 96-97 (1996).

126 Giaretta, A. A. & Cardoso, A. J. Reproductive behavior of Cycloramphus dubius Miranda-Ribeiro (Amphibia, Anura, Leptodactylidae). Revista Brasileira de Zoologia 12, 233-237 (1995).

127 Giaretta, A. A. & Facure, K. G. Reproductive ecology and behavior of Thoropa miliaris (Spix, 1824)(Anura, Leptodactylidae, Telmatobiinae). Biota Neotropica 4, 1-9 (2004).

128 Giaretta, A. A. & Facure, K. G. Terrestrial and communal nesting in Eupemphix nattereri (Anura, Leiuperidae): interactions with predators and pond structure. Journal of Natural History 40, 2577-2587 (2006).

129 Gibson, R. C. & Buley, K. R. Maternal care and obligatory oophagy in Leptodactylus fallax: A new reproductive mode in frogs. Copeia 2004, 128-135, doi:10.1643/ce-02-091r2 (2004).

130 Girish, S. & Saidapur, S. K. Mating and nesting behaviour, and early development in the tree frog Polypedates maculatus. Current science 76, 91-92 (1999).

131 Glaw, F. & Vences, M. A field guide to the amphibians and reptiles of Madagascar. (Vences & Glaw Verlag, 2007).

132 Glos, J. & Linsenmair, K. E. Descriptions of the tadpoles of Aglyptodactylus laticeps and Aglyptodactylus securifer from western Madagascar, with notes on life history and ecology. Journal of Herpetology 38, 131-136 (2004).

133 Gluesenkamp, A. G. & Acosta, N. Sexual dimorphism in Osornophryne guacamayo with notes on natural history and reproduction in the species. Journal of Herpetology 35, 148-151 (2001).

134 Goicoechea, O., Garrido, O. & Jorquera, B. Evidence for a trophic paternal-larval relationship in the frog Rhinoderma darwinii. Journal of Herpetology 20, 168-178 (1986).

135 Goldberg, F. J., Quinzio, S. & Vaira, M. Oviposition-site selection by the toad Melanophryniscus rubriventris in an unpredictable environment in Argentina. Canadian Journal of Zoology 84, 699-705 (2006).

136 Goldberg, J., Candioti, F. V. & Akmentins, M. S. Direct-developing frogs: ontogeny of Oreobates barituensis (Anura: Terrarana) and the development of a novel trait. Amphibia-Reptilia 33, 239-250 (2012).

137 Gomes, A. D., Moreira, R. G., Navas, C. A., Antoniazzi, M. M. & Jared, C. Review of the reproductive biology of caecilians (Amphibia, Gymnophiona). South American Journal of Herpetology 7, 191-202 (2012).

138 Gomez-Mesa, L. et al. Ecological and reproductive aspects of Aparasphenodon brunoi (Anura: Hylidae) in an ombrophilous forest area of the Atlantic Rainforest biome, Brazil. Zoologia (Curitiba) 34, e20477 (2017).

139 Gomez‐Mestre, I., Pyron, R. A. & Wiens, J. J. Phylogenetic analyses reveal unexpected patterns in the evolution of reproductive modes in frogs. Evolution 66, 3687-3700 (2012).

140 Gorman, T. A., Powell, S. D., Jones, K. C. & Haas, C. A. Microhabitat characteristics of egg deposition sites used by reticulated flatwoods salamanders. Herpetological Conservation and Biology 9, 543-550 (2014).

141 Gower, D. J., Giri, V., Dharne, M. S. & Shouche, Y. S. Frequency of independent origins of viviparity among caecilians (Gymnophiona): evidence from the first ‘live‐bearing’ Asian amphibian. Journal of Evolutionary Biology 21, 1220-1226 (2008).

142 Gower, D. J., Kouete, M. T., Doherty-Bone, T. M., Ndeme, E. S. & Wilkinson, M. Rediscovery, natural history, and conservation status of Idiocranium russeli Parker, 1936 (Amphibia: Gymnophiona: Indotyphlidae). Journal of Natural History 49, 233-253 (2015).

143 Grandison, A. G. C. The occurrence of Nectophrynoides (Anura Bufonidae) in Ethiopia. A new concept of the genus with a description of a new species. Monitore Zoologico Italiano 11, 119-172 (1978).

144 Grant, T. On the identities of Colostethus inguinalis (Cope, 1868) and C. panamensis (Dunn, 1933), with comments on C. latinasus (Cope, 1863) (Anura : Dendrobatidae). American Museum Novitates 3444, 1-24, doi:10.1206/0003-0082(2004)444<0001:otioci>2.0.co;2 (2004).

145 Grant, T., Acosta, A. & Rada, M. A name for the species of Allobates (Anura : Dendrobatoidea : Aromobatidae) from the Magdalena Valley of Colombia. Copeia 2007, 844-854, doi:10.1643/0045-8511(2007)7[844:anftso]2.0.co;2 (2007).

146 Grant, T. & Castro, F. The cloud forest Colostethus (Anura, Dendrobatidae) of a region of the Cordillera Occidental of Colombia. Journal of Herpetology 32, 378-392 (1998).

147 Grant, T. et al. Phylogenetic systematics of dart-poison frogs and their relatives (Amphibia: Athesphatanura: Dendrobatidae) Bulletin of the American Museum of Natural History 299, 1-262, doi:10.1206/0003-0090(2006)299[1:PSODFA]2.0.CO;2 (2006).

148 Grant, T. et al. Phylogenetic systematics of dart-poison frogs and their relatives revisited (Anura: Dendrobatoidea). South American Journal of Herpetology 12, S1-S90, doi:10.2994/SAJH-D-17-00017.1 (2017).

149 Gunther, R. Derived reproductive modes in New Guinean anuran amphibians and description of a new species with paternal care in the genus Callulops (Microhylidae). Journal of Zoology 268, 153-170, doi:10.1111/j.1469-7998.2005.00007.x (2006).

150 Gunther, R., Richards, S. J., Bickford, D. & Johnston, G. R. A new egg-guarding species of Oreophryne (Amphibia, Anura, Microhylidae) from southern Papua New Guinea. Zoosystematics and Evolution 88, 223-230, doi:10.1002/zoos.201200019 (2012).

151 Gururaja, K. V. Novel reproductive mode in a torrent frog Micrixalus saxicola (Jerdon) from the Western Ghats, India. Zootaxa 2642, 45-52 (2010).

152 Gururaja, K. V., Dinesh, K. P., Palot, M. J., Radhakrishnan, C. & Ramachandra, T. V. A new species of Philautus Gistel (Amphibia: Anura: Rhacophoridae) from southern Western Ghats, India. Zootaxa 1621, 1-16 (2007).

153 Gururaja, K. V., Dinesh, K. P., Priti, H. & Ravikanth, G. Mud-packing frog: A novel breeding behaviour and parental care in a stream dwelling new species of Nyctibatrachus (Amphibia, Anura, Nyctibatrachidae). Zootaxa 3796, 33-61, doi:10.11646/zootaxa.3796.1.2 (2014).

154 Gururaja, K. V. & Ramachandra, T. V. Developmental mode in white-nosed shrub frog Philautus cf. leucorhinus. Current Science 90, 450-454 (2006).

155 Haddad, C. F. B., Faivovich, J. & Garcia, P. C. A. The specialized reproductive mode of the treefrog Aplastodiscus perviridis (Anura: Hylidae). Amphibia-Reptilia 26, 87-92 (2005).

156 Haddad, C. F. B. & Martins, M. Four species of Brazilian poison frogs related to Epipedobates pictus (Dendrobatidae): taxonomy and natural history observations. Herpetologica 50, 282-295 (1994).

157 Haddad, C. F. B. & Prado, C. P. A. Reproductive modes in frogs and their unexpected diversity in the Atlantic Forest of Brazil. BioScience 55, 207-217 (2005).

158 Haddad, C. F. B. & Sawaya, R. J. Reproductive modes of atlantic forest hylid frogs: A general overview and the description of a new mode. Biotropica 32, 862-871 (2000).

159 Hairston, N. G. Growth, survival and reproduction of Plethodon jordani: trade-offs between selective pressures. Copeia 1983, 1024-1035 (1983).

160 Halloy, M. & Fiaño, J. M. Oviposition site selection in Pleurodema borellii (Anura: Leptodactylidae) may be influenced by tadpole presence. Copeia 2000, 606-609 (2000).

161 Hamann, M. I., Kehr, A. I., González, C. E., Duré, M. I. & Schaefer, E. F. Parasite and reproductive features of Scinax nasicus (Anura: Hylidae) from a South American subtropical area. Interciencia 34, 214-218 (2009).

162 Hampton, P. M. & Otto, K. L. Notes on the reproductive biology of the glass frog Centrolene bacatum (Anura: Centrolenidae). Phyllomedusa: Journal of Herpetology 13, 137-140 (2014).

163 Han, X. & Fu, J. Does life history shape sexual size dimorphism in anurans? A comparative analysis. BMC evolutionary biology 13, 1-11 (2013).

164 Harper, E. B. et al. Field guide to the amphibians of the Eastern Arc Mountains and Coastal Forests of Tanzania and Kenya. 320 (Camerapix Publishers International, 2010).

165 Hase, K. & Shimada, M. Female polyandry and size-assortative mating in isolated local populations of the Japanese common toad Bufo japonicus. Biological Journal of the Linnean Society 113, 236-242 (2014).

166 Hasumi, M. Reproductive behavior of the salamander Hynobius nigrescens: monopoly of egg sacs during scramble competition. Journal of Herpetology 28, 264-267 (1994).

167 Hasumi, M. Social interactions during the aquatic breeding phase of the family Hynobiidae (Amphibia: Caudata). acta ethologica 18, 243-253 (2015).

168 Hauselberger, K. F. & Alford, R. A. Effects of season and weather on calling in the Australian microhylid frogs Austrochaperina robusta and Cophixalus ornatus. Herpetologica 61, 349-363 (2005).

169 Hayes, M. P. Nest structure and attendance on the stream-dwelling frog, Eleutherodactylus angelicus. Journal of Herpetology 19, 168-169 (1985).

170 Hedges, S. B., Thomas, R. & Franz, R. A new species of Eleutherodactylus (Anura, Leptodactylidae) from the Massif de la Hotte, Haiti. Copeia 1987, 943-949 (1987).

171 Heisler, N., Forcht, G., Ultsch, G. R. & Anderson, J. F. Acid-base regulation in response to environmental hypercapnia in two aquatic salamanders, Siren lacertina and Amphiuma means. Respiration Physiology 49, 141-158 (1982).

172 Herrington, R. E. & Larsen Jr, J. H. Reproductive biology of the Larch Mountain salamander (Plethodon larselli). Journal of Herpetology 21, 48-56 (1987).

173 Heyer, W. R. & Crombie, R. I. Natural history notes on Craspedoglossa stejnegeri and Thoropa petropolitana (Amphibia: Salientia, Leptodactylidae). Journal of the Washington Academy of Sciences 69, 17-20 (1979).

174 Heying, H. Reproductive limitation by oviposition site in a treehole breeding Madagascan poison frog (Mantella laevigata). Miscellaneous Publications Museum of Zoology, University of Michigan 193, 23-30 (2004).

175 Heying, H. E. Social and reproductive behaviour in the Madagascan poison frog, Mantella laevigata, with comparisons to the dendrobatids. Animal Behaviour 61, 567-577 (2001).

176 Hillis, D. M., Hillis, A. M. & Martin, R. F. Reproductive ecology and hybridization of the endangered Houston toad (Bufo houstonensis). Journal of Herpetology 18, 56-72 (1984).

177 Hirschfeld, M. & Rödel, M.-O. Variable reproductive strategies of an African savanna frog, Phrynomantis microps (Amphibia, Anura, Microhylidae). Journal of Tropical Ecology 27, 601-609 (2011).

178 Ho, C. T., Lathrop, A., Murphy, R. W. & Orlov, N. A redescription of Vibrissaphora ailaonica with a new record in Vietnam. Russian Journal of Herpetology 6, 48-54 (1999).

179 Hödl, W. Reproductive diversity in Amazonian lowland frogs. Fortschritte der Zoologie 38, 41-60 (1990).

180 Hoffmann, H. Observations on behaviour and parental care of Leptodactylus melanonotus (Hallowell) in Costa Rica. Salamandra 42, 109-116 (2006).

181 Hollowell, T. & Reynolds, R. P. Checklist of the terrestrial vertebrates of the Guiana Shield. Bulletin of the Biological Society of Washington 13, 1-98 (2005).

182 Hoser, R. T. & Game, C. Australian reptiles & frogs. 238 (Pierson & Co, 1989).

183 Hoskin, C. J. Australian microhylid frogs (Cophixalus and Austrochaperina): phylogeny, taxonomy, calls, distributions and breeding biology. Australian Journal of Zoology 52, 237-269 (2004).

184 Hossie, T. J., Hassall, C., Knee, W. & Sherratt, T. N. Species with a chemical defence, but not chemical offence, live longer. Journal of Evolutionary Biology 26, 1598-1602 (2013).

185 Houck, L. D. Reproductive biology of a neotropical salamander, Bolitoglossa rostrata. Copeia 1977, 70-83 (1977).

186 Hutchins, M., Duellman, W. E. & Schlager, N. Vol. 6 507 (Gale Group, Farmington Hills, MI, 2003).

187 Inger, R. F. The systematics and zoogeography of the Amphibia of Borneo. Fieldiana: Zoology 52, 1-402 (1966).

188 Inger, R. F. & Voris, H. K. Taxonomic status and reproductive biology of Bornean tadpole-carrying frogs. Copeia 1988, 1060-1061 (1988).

189 Inger, R. F., Voris, H. K. & Walker, P. Larval transport in a Bornean ranid frog. Copeia 1986, 523-525 (1986).

190 Iskandar, D. T., Evans, B. J. & McGuire, J. A. A novel reproductive mode in frogs: a new species of fanged frog with internal fertilization and birth of tadpoles. PLoS One 9, e115884 (2014).

191 IUCN. The IUCN Red List of Threatened Species. Version 2018-1, 2018).

192 Jadhav, B. V. Ecology of Ichthyophis bombayensis (Gymnophiona: Amphibia) from Koyana region, Maharashtra, India. Biological Forum 2, 14-17 (2010).

193 Jared, C., Antoniazzi, M. M., Katchburian, E., Toledo, R. C. & Freymüller, E. Some aspects of the natural history of the casque-headed tree frog Corythomantis greeningi Boulenger (Hylidae). Annales des Sciences Naturelles - Zoologie et Biologie Animale 20, 105-115 (1999).

194 Jockusch, E. L. & Mahoney, M. J. Communal oviposition and lack of parental care in Batrachoseps nigriventris (Caudata: Plethodontidae) with a discussion of the evolution of breeding behavior in plethodontid salamanders. Copeia 1997, 697-705 (1997).

195 Joglar, R. L., Burrowes, P. A. & Rios, N. in Contributions to West Indian Herpetology: A Tribute to Albert Schwartz (eds Robert Powell & Robert W Henderson) 251-258 (Society for the Study of Amphibians and Reptiles, 1996).

196 Juncá, F. A., Altig, R. & Gascon, C. Breeding biology of Colostethus stepheni, a dendrobatid frog with a nontransported nidicolous tadpole. Copeia 1994, 747-750 (1994).

197 Jungfer, K.-H. Beitrag zur Kenntnis von Dendrobates speciosus. Salamandra 21, 263-280 (1985).

198 Jungfer, K.-H. Reproduction and parental care of the coronated treefrog, Anotheca spinosa (Steindachner, 1864)(Anura: Hylidae). Herpetologica 52, 25-32 (1996).

199 Jungfer, K. H. et al. Systematics of spiny‐backed treefrogs (Hylidae: Osteocephalus): an Amazonian puzzle. Zoologica Scripta 42, 351-380 (2013).

200 Jungfer, K.-H., Ron, S., Seipp, R. & Almendáriz, A. Two new species of hylid frogs, genus Osteocephalus, from Amazonian Ecuador. Amphibia-Reptilia 21, 327-340 (2000).

201 Jungfer, K.-H. & Schiesari, L. C. Description of a central Amazonian and Guianan tree frog, genus Osteocephalus (Anura, Hylidae), with oophagous tadpoles. Alytes 13, 1-13 (1995).

202 Jungfer, K.-H. & Weygoldt, P. Biparental care in the tadpole-feeding Amazonian treefrog Osteocephalus oophagus. Amphibia-Reptilia 20, 235-249 (1999).

203 Jungfer, K.-H., Weygoldt, P. & Juraske, N. Dendrobates vicentei, ein neuer Pfeilgiftfrosch aus Zentral–Panama. Herpetofauna 18, 17-26 (1996).

204 Kadadevaru, G. G. & Kanamadi, R. D. Courtship and nesting behaviour of the Malabar gliding frog, Rhacophorus malabaricus (Jerdon, 1870). Current Science 79, 378-380 (2000).

205 Kaefer, I. L., Both, C. & Cechin, S. Z. Breeding biology of the rapids frog Limnomedusa macroglossa (Anura: Cycloramphidae) in southern Brazil. Journal of Natural History 43, 1195-1206 (2009).

206 Kaiser, H. & Altig, R. The atypical tadpole of the dendrobatid frog, Colostethus chalcopis, from Martinique, French Antilles. Journal of Herpetology 28, 374-378 (1994).

207 Kaiser, H., Coloma, L. A. & Gray, H. M. A new species of Colostethus (Anura: Dendrobatidae) from Martinique, French Antilles. Herpetologica 50, 23-32 (1994).

208 Kaiser, H., Green, D. M. & Schmid, M. Systematics and biogeography of Eastern Caribbean frogs (Leptodactylidae: Eleutherodactylus), with the description of a new species from Dominica. Canadian Journal of Zoology 72, 2217-2237 (1994).

209 Kakegawa, M. & Hasumi, M. Effects of controlled water temperatures on oviposition in a lotic‐breeding and externally fertilizing salamander (Hynobius kimurae). River Research and Applications 33, 1036-1043 (2017).

210 Kam, Y.-C., Chen, Y.-H., Chen, T.-C. & Tsai, I.-R. Maternal brood care of an arboreal breeder, Chirixalus eiffingeri (Anura: Rhacophoridae) from Taiwan. Behaviour 137, 137-151 (2000).

211 Kam, Y.-C., Chuang, Z.-S. & Yen, C.-F. Reproduction, oviposition-site selection, and tadpole oophagy of an arboreal nester, Chirixalus eiffingeri (Rhacophoridae), from Taiwan. Journal of Herpetology 30, 52-59 (1996).

212 Kamei, R. G., Gower, D. J., Wilkinson, M. & Biju, S. D. Systematics of the caecilian family Chikilidae (Amphibia: Gymnophiona) with the description of three new species of Chikila from northeast India. Zootaxa 3666, 401-435 (2013).

213 Kamei, R. G. et al. Discovery of a new family of amphibians from northeast India with ancient links to Africa. Proceedings of the Royal Society of London B: Biological Sciences 279, 2396-2401 (2012).

214 Kaminsky, S. K., Linsenmair, K. E. & Grafe, T. U. Reproductive timing, nest construction and tadpole guidance in the African pig-nosed frog, Hemisus marmoratus. Journal of Herpetology 33, 119-123 (1999).

215 Katsuren, S., Tanaka, S. & Ikehara, S. A brief observation on the breeding site and eggs of a frog, Rana ishikawae (Stejneger) in Okinawa Island. Ecological Studies of Nature Conservation of the Ryukyu Islands 3, 49-54 (1977).

216 Kern, M. M., Nassar, A. A., Guzy, J. C. & Dorcas, M. E. Oviposition site selection by spotted salamanders (Ambystoma maculatum) in an isolated wetland. Journal of Herpetology 47, 445-449 (2013).

217 Khattak, S. et al. Optimized axolotl (Ambystoma mexicanum) husbandry, breeding, metamorphosis, transgenesis and tamoxifen-mediated recombination. Nature Protocols 9, 529-540 (2014).

218 Khongwir, S., Hooroo, R. N. K. & Dutta, S. K. Breeding and nesting behaviour of Rhacophorus maximus (Anura: Rhacophoridae) in Meghalaya, North East India. Current Science 110, 1102-1105 (2016).

219 Kidov, A. A., Matushkina, K. A., Uteshev, V. K., Timoshina, A. L. & Kovrina, E. G. The first captive breeding of the Eichwald's toad (Bufo eichwaldi). Russian Journal of Herpetology 21, 40-46 (2014).

220 Kluge, A. G. The life history, social organization, and parental behavior of Hyla rosenbergi Boulenger, a nest-building gladiator frog. Miscellaneous Publications Museum of Zoology, University of Michigan 160, 1-170 (1981).

221 Köhler, F. & Günther, R. The radiation of microhylid frogs (Amphibia: Anura) on New Guinea: a mitochondrial phylogeny reveals parallel evolution of morphological and life history traits and disproves the current morphology-based classification. Molecular Phylogenetics and Evolution 47, 353-365 (2008).

222 Kohler, J., Glaw, F. & Vences, M. Notes on the reproduction of Rhombophryne (Anura: Microhylidae) at Nosy Be, northern Madagascar. Revue fr. Aquariol. 24, 53-54 (1997).

223 Kok, D., du Preez, L. H. & Channing, A. Channel construction by the African bullfrog: another anuran parental care strategy. Journal of Herpetology 23, 435-437 (1989).

224 Kok, D. J. & Seaman, M. T. Natalobatrachus bonebergi (Anura: Ranidae): aspects of early development and adult size. South African Journal of Zoology 23, 238-241 (1988).

225 Kok, P. J. R. A redescription of Anomaloglossus praderioi (La Marca, 1998)(Anura: Aromobatidae: Anomaloglossinae), with description of its tadpole and call. Papéis Avulsos de Zoologia (São Paulo) 50, 51-68 (2010).

226 Kok, P. J. R., Sambhu, H., Roopsind, I., Lenglet, G. L. & Bourne, G. R. A new species of Colostethus (Anura: Dendrobatidae) with maternal care from Kaieteur National Park, Guyana. Zootaxa 1238, 35-61 (2006).

227 Kouete, M. T., Wilkinson, M. & Gower, D. J. First reproductive observations for Herpele Peters, 1880 (Amphibia: Gymnophiona: Herpelidae): evidence of extended parental care and maternal dermatophagy in H. squalostoma (Stutchbury, 1836). ISRN Zoology 2012, 1-7 (2012).

228 Krenz, J. D. & Sever, D. M. Mating and oviposition in paedomorphic Ambystoma talpoideum precedes the arrival of terrestrial males. Herpetologica 51, 387-393 (1995).

229 Krügel, P. & Richter, S. Syncope antenori: a bromeliad breeding frog with free-swimming, nonfeeding tadpoles (Anura, Microhylidae). Copeia 1995, 955-963 (1995).

230 Kupfer, A., Maxwell, E., Reinhard, S. & Kuehnel, S. The evolution of parental investment in caecilian amphibians: a comparative approach. Biological Journal of the Linnean Society 119, 4-14 (2016).

231 Kupfer, A. et al. Parental investment by skin feeding in a caecilian amphibian. Nature 440, 926-929 (2006).

232 Kupfer, A., Wilkinson, M., Gower, D. J., Müller, H. & Jehle, R. Care and parentage in a skin‐feeding caecilian amphibian. Journal of Experimental Zoology Part A: Ecological Genetics and Physiology 309, 460-467 (2008).

233 Kusano, T. Breeding and egg survival of a population of a salamander, Hynobius nebulosus tokyoensis Tago. Researches on Population Ecology 21, 181-196 (1980).

234 Kuzmin, S. L., Dasgupta, R. & Smirina, E. M. Ecology of the Himalayan newt (Tylototriton verrucosus) in Darjeeling himalayas, India. Russian Journal of Herpetology 1, 69-76 (1994).

235 Lamotte, M. & Lescure, J. Tendances adaptatives a l’affranchissement du milieu aquatique chez les amphibiens anoures. La Terre et la Vie 31, 225-311 (1977).

236 Landestoy, M. Observations on the breeding behaviour of the Hispaniolan green treefrog, Hypsiboas heilprini. IRCF Reptiles & Amphibians 20, 160-165 (2013).

237 Lannoo, M. J. Amphibian declines: the conservation status of United States species. (University of California Press, 2005).

238 Lannoo, M. J., Townsend, D. S. & Wassersug, R. J. Larval life in the leaves: arboreal tadpole types, with special attention to the morphology, ecology, and behavior of the oophagous Osteopilus brunneus (Hylidae) larva. Fieldiana Zoology 38, 1-31 (1987).

239 Lauck, B., Swain, R. & Barmuta, L. Breeding site characteristics regulating life history traits of the brown tree frog, Litoria ewingii. Hydrobiologia 537, 135-146 (2005).

240 Lee, S., Zippel, K., Ramos, L. & Searle, J. Captive‐breeding programme for the Kihansi spray toad Nectophrynoides asperginis at the Wildlife Conservation Society, Bronx, New York. International Zoo Yearbook 40, 241-253 (2006).

241 Lehtinen, R. M. Parental care and reproduction in two species of Mantidactylus (Anura: Mantellidae). Journal of Herpetology 37, 766-768 (2003).

242 Lehtinen, R. M. The natural history of two plant-breeding frogs from Madagascar, Guibemantis bicalcaratus and G. punctatus (Anura: Mantellidae). Salamandra 45, 39-49 (2009).

243 Lehtinen, R. M., Lannoo, M. J. & Wassersug, R. J. Phytotelm-breeding Anurans: past, present and future research. Miscellaneous Publications Museum of Zoology, University of Michigan 193, 1-9 (2004).

244 Lehtinen, R. M. & Nussbaum, R. A. in Reproductive biology and phylogeny of Anura Vol. 2 (ed Barrie GM Jamieson) 343-386 (Science Publishers, Inc., 2003).

245 Lemos-Espinal, J. A., Smith, G. R., Ruíz, Á. H. & Ayala, R. M. Natural history, phenology, and stream use of Hyla plicata from the Arroyo Los Axolotes, State of México, Mexico. Current Herpetology 35, 8-13 (2016).

246 Li, J.-t. et al. New insights to the molecular phylogenetics and generic assessment in the Rhacophoridae (Amphibia: Anura) based on five nuclear and three mitochondrial genes, with comments on the evolution of reproduction. Molecular Phylogenetics and Evolution 53, 509-522 (2009).

247 Liao, W. B. & Lu, X. Breeding behaviour of the Omei tree frog Rhacophorus omeimontis (Anura: Rachophoridae) in a subtropical montane region. Journal of Natural History 44, 2929-2940 (2010).

248 Liedtke, H. C., Gower, D. J., Wilkinson, M. & Gomez-Mestre, I. Macroevolutionary shift in the size of amphibian genomes and the role of life history and climate. Nature ecology & evolution 2, 1792-1799 (2018).

249 Liedtke, H. C., Müller, H., Hafner, J., Nagel, P. & Loader, S. P. Interspecific patterns for egg and clutch sizes of African Bufonidae (Amphibia: Anura). Zoologischer Anzeiger-A Journal of Comparative Zoology 253, 309-315 (2014).

250 Lima, A. P., Caldwell, J. P. & Biavati, G. M. Territorial and reproductive behavior of an Amazonian dendrobatid frog, Colostethus caeruleodactylus. Copeia 2002, 44-51 (2002).

251 Lima, A. P., Caldwell, J. P. & Strussmann, C. Redescription of Allobates brunneus (Cope) 1887 (Anura: Aromobatidae: Allobatinae), with a description of the tadpole, call, and reproductive behavior. Zootaxa 1988, 1-16 (2009).

252 Lima, A. P. & Keller, C. Reproductive characteristics of Colostethus marchesianus from its type locality in Amazonas, Brazil. Journal of Herpetology 37, 754-757 (2003).

253 Liu, C.-c. Amphibians of western China. Fieldiana: Zoology Memoirs. Vol. 2 (Chicago Natural History Museum, 1950).

254 Loader, S. P., Wilkinson, M., Gower, D. J. & Msuya, C. A. A remarkable young Scolecomorphus vittatus (Amphibia: Gymnophiona: Scolecomorphidae) from the North Pare Mountains, Tanzania. Journal of Zoology 259, 93-101 (2003).

255 Lunghi, E. et al. Nesting of cave salamanders (Hydromantes flavus and H. italicus) in natural environments. Salamandra 50, 105-109 (2014).

256 Lutz, B. Anfíbios novos e raros das serras costeiras do Brasil. Memórias do Instituto Oswaldo Cruz 56, 373-399 (1958).

257 Lynch, J. D., Ruiz, P. M. & Rueda, J. V. Notes on the distribution and reproductive biology of Centrolene geckoideum Jimenez de la Espada in Colombia and Ecuador (Amphibia: Centrolenidae). Studies on Neotropical Fauna and Environment 18, 239-243 (1983).

258 Maciel, A. O., Gomes, J. O., Costa, J. C. L. & Andrade, G. V. Diet, microhabitat use, and an analysis of sexual dimorphism in Caecilia gracilis (Amphibia: Gymnophiona: Caeciliidae) from a riparian forest in the Brazilian Cerrado. Journal of Herpetology 46, 47-50 (2012).

259 Maciel, A. O. & Hoogmoed, M. S. Taxonomy and distribution of caecilian amphibians (Gymnophiona) of Brazilian Amazonia, with a key to their identification. Zootaxa 2984, 1-53 (2011).

260 Magnusson, W. E., Lima, A. P., Hero, J.-M. & de Araújo, M. C. The rise and fall of a population of Hyla boans: reproduction in a Neotropical gladiator frog. Journal of Herpetology 33, 647-656 (1999).

261 Malone, J. H. Reproduction in three species of Smilisca from Costa Rica. Journal of Herpetology 38, 27-35 (2004).

262 Malonza, P. K. & Measey, G. J. Life history of an African caecilian: Boulengerula taitanus Loveridge 1935 (Amphibia Gymnophiona Caeciilidae). Tropical Zoology 18, 49-66 (2005).

263 Marinetti, G. V., T, B. J. & Cattieu, K. Lipid composition of eggs and tadpoles of Agalychnis dacnicolor and Rana pipiens. Comparative Biochemistry and Physiology Part B: Comparative Biochemistry 70, 779-782 (1981).

264 Martins, M., Pombal, J. P. & Haddad, C. F. B. Escalated aggressive behaviour and facultative parental care in the nest building gladiator frog, Hyla faber. Amphibia-Reptilia 19, 65-73 (1998).

265 Marvin, G. A. Life history and population characteristics of the salamander Plethodon kentucki with a review of Plethodon life histories. American Midland Naturalist 136, 385-400 (1996).

266 McDiarmid, R. W. & Gorzula, S. Aspects of the reproductive ecology and behavior of the tepui toads, genus Oreophrynella (Anura, Bufonidae). Copeia 1989, 445-451 (1989).

267 Measey, G. J. Are caecilians rare? An east African perspective. Journal of East African Natural History 93, 1-21 (2004).

268 Measey, G. J. & Di-Bernardo, M. Estimating juvenile abundance in a population of the semiaquatic caecilian, Chthonerpeton indistinctum (Amphibia: Gymnophiona: Typhlonectidae), in southern Brazil. Journal of Herpetology 37, 371-373 (2003).

269 Measey, G. J., Gower, D. J., Oommen, O. V. & Wilkinson, M. A mark–recapture study of the caecilian amphibian Gegeneophis ramaswamii (Amphibia: Gymnophiona: Caeciliidae) in southern India. Journal of Zoology 261, 129-133 (2003).

270 Measey, G. J. & Tinsley, R. C. Mating behavior of Xenopus wittei (Anura: Pipidae). Copeia 1997, 601-609 (1997).

271 Meegaskumbura, M. et al. Patterns of reproductive‐mode evolution in Old World tree frogs (Anura, Rhacophoridae). Zoologica Scripta 44, 509-522 (2015).

272 Mendelson III, J. R. et al. Spectacular new gliding species of Ecnomiohyla (Anura: Hylidae) from central Panama. Journal of Herpetology 42, 750-759 (2008).

273 Mendelson III, J. R., Ustach, P. C. & De Oca, A. N. M. Description of the tadpole of Bufo tutelarius, natural history notes on the Bufo valliceps group, and a key to the tadpoles of the group. Journal of Herpetology 33, 324-328 (1999).

274 Menegon, M., Salvidio, S. & Loader, S. Five new species of Nectophrynoides Noble 1926 (Amphibia Anura Bufonidae) from the Eastern Arc Mountains, Tanzania. Tropical Zoology 17, 97-121 (2004).

275 Menin, M., Lima, A. P. & Rodrigues, D. J. The tadpole of Leptodactylus pentadactylus (Anura: Leptodactylidae) from central Amazonia. Zootaxa 2508, 65-68 (2010).

276 Menin, M., Rodrigues, D. & Lima, A. Clutches, tadpoles and advertisement calls of Synapturanus mirandaribeiroi and S. cf. salseri in Central Amazonia, Brazil. The Herpetological Journal 17, 86-91 (2007).

277 Miaud, C. Predation on newt eggs (Triturus alpestris and T. helveticus): identification of predators and protective role of oviposition behaviour. Journal of Zoology 231, 575-582 (1993).

278 Miaud, C. Role of wrapping behavior on egg survival in three species of Triturus (Amphibia: Urodela). Copeia 1994, 535-537 (1994).

279 Michaels, C. J. et al. Breeding and rearing the critically endangered lake Oku clawed frog (Xenopus longipes Loumont and Kobel 1991). Amphibian & Reptile Conservation 9, 100-110 (2015).

280 Milanovich, J. R., Trauth, S. E., Saugey, D. A. & Jordan, R. R. Fecundity, reproductive ecology, and influence of precipitation on clutch size in the western slimy salamander (Plethodon albagula). Herpetologica 62, 292-301 (2006).

281 Minter, L. R. et al. (SI/MAB Series #9, Smithsonian Institution, Washington DC, 2004).

282 Mirabile, M., Melletti, M., Venchi, A. & Bologna, M. A. The reproduction of the Apennine yellow-bellied toad (Bombina pachypus) in central Italy. Amphibia-Reptilia 30, 303-312 (2009).

283 Mitchell, N. J. Low tolerance of embryonic desiccation in the terrestrial nesting frog Bryobatrachus nimbus (Anura: Myobatrachinae). Copeia 2002, 364-373 (2002).

284 Mitchell, N. J. & Seymour, R. S. Effects of temperature on energy cost and timing of embryonic and larval development of the terrestrially breeding moss frog, Bryobatrachus nimbus. Physiological and Biochemical Zoology 73, 829-840 (2000).

285 Moen, D. S. & Wiens, J. J. Microhabitat and climatic niche change explain patterns of diversification among frog families. The American Naturalist 190, 29-44 (2017).

286 Montanarin, A., Kaefer, I. L. & Lima, A. P. Courtship and mating behaviour of the brilliant-thighed frog Allobates femoralis from Central Amazonia: implications for the study of a species complex. Ethology Ecology & Evolution 23, 141-150 (2011).

287 Montero, I., Reichle, S. & Kupfer, A. Observations on the reproductive ecology of Siphonops paulensis Boettger, 1892 (Gymnophiona: Caeciliidae) in Bolivia. Salamandra 41, 91-94 (2005).

288 Moore, J. A. The frogs of eastern New South Wales. Bulletin of the American Museum of Natural History 121, 149-386 (1961).

289 Moravec, J. et al. A new species of Osteocephalus (Anura: Hylidae) from Amazonian Bolivia: first evidence of tree frog breeding in fruit capsules of the Brazil nut tree. Zootaxa 2215, 37-54 (2009).

290 Moreno, M. C. D., Guyer, C. & Bailey, M. A. Distribution and population biology of the Black Warrior Waterdog, Necturus alabamensis. Southeastern Naturalist 5, 69-84 (2006).

291 Moser, G. The evolution of phytotelmata-breeding anurans: Breeding and calling site characteristics of the two tree hole breeding frogs Trachycephalus resinifictrix and T. hadroceps (Anura: Hylidae) Magister der Naturwissenschaften thesis, Universität Wien, (2010).

292 Moura, M. R. d., Motta, A. P. & Feio, R. N. An unusual reproductive mode in Hypsiboas (Anura: Hylidae). Zoologia (Curitiba) 28, 142-144 (2011).

293 Muedeking, M. H. & Heyer, W. R. Descriptions of eggs and reproductive patterns of Leptodactylus pentadactylus (Amphibia: Leptodactylidae). Herpetologica 32, 137-139 (1976).

294 Müller, H., Loader, S. P., Ngalason, W., Howell, K. M. & Gower, D. J. Reproduction in Brevicipitid frogs (Amphibia: Anura: Brevicipitidae): Evidence from Probreviceps m. macrodactylus. Copeia 2007, 726-733 (2007).

295 Müller, H., Wilkinson, M., Loader, S. P., Wirkner, C. S. & Gower, D. J. Morphology and function of the head in foetal and juvenile Scolecomorphus kirkii (Amphibia: Gymnophiona: Scolecomorphidae). Biological journal of the Linnean Society 96, 491-504 (2009).

296 Myers, C. W., Daly, J. W. & Martínez, V. An arboreal poison frog (Dendrobates) from western Panama. American Museum Novitates 2783, 1-20 (1984).

297 Nali, R. C. & Prado, C. P. A. Habitat use, reproductive traits and social interactions in a stream-dweller treefrog endemic to the Brazilian Cerrado. Amphibia-Reptilia 33, 337-347 (2012).

298 Nishikawa, K., Matsui, M., Imbun, P. Y., Lakim, M. B. & Mohamed, M. Field observation of egg brooding in the caecilian Caudacaecilia asplenia from Sabah, Malaysia (Amphibia: Gymnophiona: Ichthyophiidae). The Raffles Bulletin of Zoology 56, 205-208 (2008).

299 Noble, G. K. The adaptive modifications of the arboreal tadpoles of Hoplophryne and the torrent tadpoles of Staurois. Bulletin of the American Museum of Natural History LVIII, 291-334 (1929).

300 Norris, K. M. & Hosie, C. A. A quantified ethogram for oviposition in Triturus newts: description and comparison of T. helveticus and T. vulgaris. Ethology 111, 357-366 (2005).

301 Nunez, J. J. & Ubeda, C. A. The tadpole of Eupsophus nahuelbutensis (Anura: Neobatrachia): external morphology, chondrocranium, and comments on its natural history. Zootaxa 2126, 58-68 (2009).

302 Nussbaum, R. A. The evolution of parental care in salamanders. Miscellaneous publications Museum of Zoology, University of Michigan 169, 1-50 (1985).

303 Nussbaum, R. A. Parental care and egg size in salamanders: an examination of the safe harbor hypothesis. Researches on Population Ecology 29, 27-44 (1987).

304 Nussbaum, R. A. in Reproductive biology and phylogeny of Urodela (ed Barrie GM Jamieson) 527-612 (CRC Press, 2003).

305 Ohdachi, S. Growth, metamorphosis, and gape-limited cannibalism and predation on tadpoles in larvae of salamanders Hynobius retardatus. Zoological Science 11, 127-131 (1994).

306 Oliveira, B. F., São-Pedro, V. A., Santos-Barrera, G., Penone, C. & Costa, G. C. AmphiBIO, a global database for amphibian ecological traits. Scientific Data 4, 170123, doi:10.1038/sdata.2017.123 (2017).

307 Oneto, F., Ottonello, D., Pastorino, M. V. & Salvidio, S. Posthatching parental care in salamanders revealed by infrared video surveillance. Journal of Herpetology 44, 649-653 (2010).

308 O'Reilly, J. C., Fenolio, D., Rania, L. C. & Wilkinson, M. Altriciality and extended parental care in the West African caecilian Geotrypetes seraphini (Gymnophiona: Caeciliidae). American Zoologist 38, 187A (1998).

309 Osikowski, A. & Rafinski, J. Multiple insemination increases reproductive success of female Montandon’s newt (Triturus montandoni, Caudata, Salamandridae). Behavioral Ecology and Sociobiology 49, 145-149 (2001).

310 Ospina-Sarria, J. J., Bolívar-G, W., Mendez-Narvaez, J. & Burbano-Yandi, C. The tadpole of Nymphargus grandisonae (Anura, Centrolenidae) from Valle del Cauca, Colombia. South American Journal of Herpetology 6, 79-86 (2011).

311 Ovaska, K. & Rand, A. S. Courtship and reproductive behavior of the frog Eleutherodactylus diastema (Anura: Leptodactylidae) in Gamboa, Panama. Journal of Herpetology 35, 44-50 (2001).

312 Ovaska, K. E. & Caldbeck, J. Courtship behavior and vocalizations of the frogs Eleutherodactylus antillensis and E. cochranae on the British Virgin Islands. Journal of Herpetology 31, 149-155 (1997).

313 Owen, P. C. & Tucker, J. K. Courtship calls and behavior in two species of chorus frogs, genus Pseudacris (Anura: Hylidae). Copeia 2006, 137-144 (2006).

314 Park, D. The first observation of breeding of the Long-tailed clawed salamander, Onychodactylus fischeri, in the field. Current Herpetology 24, 7-12 (2005).

315 Passmore, N. I. Vocalizations and breeding behaviour of Ptychadena taenioscelis (Anura: Ranidae). Zoologica Africana 11, 339-347 (1976).

316 Pašukonis, A. et al. Induced parental care in a poison frog: a tadpole cross-fostering experiment. Journal of Experimental Biology, doi:10.1242/jeb.165126 (2017).

317 Peixoto, O. L., Caramaschi, U. & Freire, E. M. X. Two new species of Phyllodytes (Anura: Hylidae) from the state of Alagoas, northeastern Brazil. Herpetologica 59, 235-246 (2003).

318 Pereira, E. B., Collevatti, R. G., de Carvalho Kokubum, M. N., de Oliveira Miranda, N. E. & Maciel, N. M. Ancestral reconstruction of reproductive traits shows no tendency toward terrestriality in leptodactyline frogs. BMC Evolutionary Biology 15, 1-12 (2015).

319 Pereira, E. B. et al. Evolution of the anuran foam nest: trait conservatism and lineage diversification. Biological Journal of the Linnean Society 122, 814-823 (2017).

320 Pereyra, L. C., Lescano, J. N. & Leynaud, G. C. Breeding-site selection by red-belly toads, Melanophryniscus stelzneri (Anura: Bufonidae), in Sierras of Córdoba, Argentina. Amphibia-Reptilia 32, 105-112 (2011).

321 Pérez, O. D., Lai, N. B., Buckley, D., del Pino, E. M. & Wake, M. H. The morphology of prehatching embryos of Caecilia orientalis (Amphibia: Gymnophiona: Caeciliidae). Journal of Morphology 270, 1492-1502 (2009).

322 Perotti, M. G. Modos reproductivos y variables reproductivas cuantitativas de un ensamble de anuros del Chaco semiárido, Salta, Argentina. Revista Chilena de Historia Natural 70, 277-288 (1997).

323 Phimmachak, S., Stuart, B. L. & Sivongxay, N. Distribution, natural history, and conservation of the Lao newt (Laotriton laoensis)(Caudata: Salamandridae). Journal of Herpetology 46, 120-128 (2012).

324 Pinto, R. M. C. & Menin, M. Aspects of the natural history of Leptodactylus knudseni Heyer, 1972 (Anura: Leptodactylidae) in a pristine forest in Central Amazonia, Brazil, with comments on ontogenetic variation of its tadpoles. Journal of Natural History 51, 2523-2534 (2017).

325 Pinya, S. & Pérez-Mellado, V. Clutch size in wild populations of Alytes muletensis. Acta Herpetologica 9, 115-117 (2014).

326 Poelman, E. H. & Dicke, M. Offering offspring as food to cannibals: oviposition strategies of Amazonian poison frogs (Dendrobates ventrimaculatus). Evolutionary Ecology 21, 215-227 (2007).

327 Poelman, E. H. & Dicke, M. Space use of Amazonian poison frogs: testing the reproductive resource defense hypothesis. Journal of Herpetology 42, 270-278 (2008).

328 Pombal Jr, J. P., Sazima, I. & Haddad, C. F. B. Breeding behavior of the pumpkin toadlet, Brachycephalus ephippium (Brachycephalidae). Journal of Herpetology 28, 516-519 (1994).

329 Portik, D. M. & Blackburn, D. C. The evolution of reproductive diversity in Afrobatrachia: A phylogenetic comparative analysis of an extensive radiation of African frogs. Evolution 70, 2017-2032 (2016).

330 Prado, C. P. A. & Haddad, C. F. B. Size-fecundity relationships and reproductive investment in female frogs in the Pantanal, south-western Brazil. The Herpetological Journal 15, 181-189 (2005).

331 Prado, C. P. A., Toledo, L. F., Zina, J. & Haddad, C. F. B. Trophic eggs in the foam nests of Leptodactylus labyrinthicus (Anura, Leptodactylidae): an experimental approach. The Herpetological Journal 15, 279-284 (2005).

332 Prado, C. P. d. A., Uetanabaro, M. & Haddad, C. F. B. Description of a new reproductive mode in Leptodactylus (Anura, Leptodactylidae), with a review of the reproductive specialization toward terrestriality in the genus. Copeia 2002, 1128-1133 (2002).

333 Prado, C. P. d. A., Uetanabaro, M. & Lopes, F. S. Reproductive strategies of Leptodactylus chaquensis and L. podicipinus in the Pantanal, Brazil. Journal of Herpetology 34, 135-139 (2000).

334 Proehl, H. & Hoedl, W. Parental investment, potential reproductive rates, and mating system in the strawberry dart-poison frog, Dendrobates pumilio. Behavioral Ecology and Sociobiology 46, 215-220 (1999).

335 Quiguango-Ubillus, A. & Coloma, L. A. Notes on behaviour, communication and reproduction in captive Hyloxalus toachi (Anura: Dendrobatidae), an Endangered Ecuadorian frog. International Zoo Yearbook 42, 78-89 (2008).

336 Quijano, F. M., Santos-Barrera, G. & Pacheco-Rodriguez, J. Eleutherodactylus fitzingeri (common rain frog). Clutch size and parental care. Herpetological Review 33, 125 (2002).

337 Quinn, H. R. & Mengden, G. Reproduction and growth of Bufo houstonensis (Bufonidae). The Southwestern Naturalist 29, 189-195 (1984).

338 Reading, C. J. & Jofré, G. M. Reproduction in the nest building vizcacheras frog Leptodactylus bufonius in central Argentina. Amphibia-Reptilia 24, 415-427 (2003).

339 Reinhard, S., Voitel, S. & Kupfer, A. External fertilisation and paternal care in the paedomorphic salamander Siren intermedia Barnes, 1826 (Urodela: Sirenidae). Zoologischer Anzeiger-A Journal of Comparative Zoology 253, 1-5 (2013).

340 Ringia, A. M. & Lips, K. R. Oviposition, early development and growth of the cave salamander, Eurycea lucifuga: surface and subterranean influences on a troglophilic species. Herpetologica 63, 258-268 (2007).

341 Ringler, E., Pašukonis, A., Ringler, M. & Huber, L. Sex-specific offspring discrimination reflects respective risks and costs of misdirected care in a poison frog. Animal behaviour 114, 173-179 (2016).

342 Ríos-López, N., Agosto-Torres, E., Hernández-Muñíz, R. M. & Cao, G. M. Natural history notes on the reproductive biology of the melodious coqui, Eleutherodactylus wightmanae (Schmidt, 1920), the whistling coqui, E. cochranae (Grant, 1932), and the mountain coqui, E. portoricensis (Schmidt, 1927)(Anura: Eleutherodactylidae), from Puerto Rico. Life: The Excitement of Biology 4, 3-10 (2016).

343 Roberts, W. E. Explosive breeding aggregations and parachuting in a Neotropical frog, Agalychnis saltator (Hylidae). Journal of Herpetology 28, 193-199 (1994).

344 Rödel, M.-O. A reproductive mode so far unknown in African ranids: Phrynobatrachus guineensis Guibe & Lamotte, 1961 breeds in tree holes. Herpetozoa 11, 19-26 (1998).

345 Rödel, M.-O. & Ernst, R. A new reproductive mode for the genus Phrynobatrachus: Phrynobatrachus alticola has nonfeeding, nonhatching tadpoles. Journal of Herpetology 36, 121-125 (2002).

346 Rödel, M.-O., Kosuch, J., Veith, M. & Ernst, R. First record of the genus Acanthixalus Laurent, 1944 from the upper Guinean rain forest, West Africa, with the description of a new species. Journal of Herpetology 37, 43-52 (2003).

347 Rodrigues, A. P., Giaretta, A. A., da Silva, D. R. & Facure, K. G. Reproductive features of three maternal-caring species of Leptodactylus (Anura: Leptodactylidae) with a report on alloparental care in frogs. Journal of Natural History 45, 2037-2047 (2011).

348 Rodrigues, D. J., Uetanabaro, M. & Lopes, F. S. Breeding biology of Phyllomedusa azurea Cope, 1862 and P. sauvagii Boulenger, 1882 (Anura) from the Cerrado, central Brazil. Journal of Natural History 41, 1841-1851 (2007).

349 Rodríguez, L. O. & Myers, C. W. A new poison frog from Manu National Park, southeastern Peru (Dendrobatidae, Epipedobates). American Museum Novitates 3068, 1-15 (1993).

350 Ron, S., Narváez, A. & Romero, G. Reproduction and spawning behavior in the frog, Engystomops pustulatus (Shreve 1941). Amphibian & Reptile Conservation 8, 25-32 (2014).

351 Rosa, G. M., Mercurio, V., Crottini, A. & Andreone, F. Explosion into the canyon: an insight into the breeding aggregation of Scaphiophryne gottlebei Busse & Böhme, 1992. North-Western Journal of Zoology 7, 329-333 (2011).

352 Rowley, J. et al. The breeding biologies of three species of treefrogs with hyperextended vocal repertoires (Gracixalus; Anura: Rhacophoridae). Amphibia-Reptilia 36, 277-285 (2015).

353 Rowley, J. J. L., Le, D. T. T., Hoang, H. D. & Altig, R. The breeding behaviour, advertisement call and tadpole of Limnonectes dabanus (Anura: Dicroglossidae). Zootaxa 3881, 195-200 (2014).

354 Roy, D. & Mushahidunnabi, M. Courtship, mating and egg-laying in Tylototriton verrucosus from the Darjeeling district of the Eastern Himalaya. Current Science 81, 693-695 (2001).

355 Russell, K. R., Gonyaw, A. A., Strom, J. D., Diemer, K. E. & Murk, K. C. Three new nests of the Columbia torrent salamander, Rhyacotriton kezeri, in Oregon with observations of nesting behavior. Northwestern Naturalist 83, 19-22 (2002).

356 Ryan, M. Egg attendance by female frogs in two species of Eleutherodactylus from Costa Rica. Herpetological Review 36, 234-235 (2005).

357 Ryan, M. J. & Barry, D. S. Competitive interactions in phytotelmata—Breeding pools of two poison-dart frogs (Anura: Dendrobatidae) in Costa Rica. Journal of Herpetology 45, 438-443 (2011).

358 Saba, N., Balwan, W. K. & Tripathi, N. K. Larval cannibalism in the Indus Valley toad, Duttaphrynus stomaticus. Bulletin of Environment, Pharmacology and Life Sciences 2, 148-150 (2013).

359 Salgado, A. L. & Guayasamin, J. M. Parental Care and Reproductive Behavior of the Minute Dappled Glassfrog (Centrolenidae: Centrolene peristictum). South American Journal of Herpetology 13, 211-219 (2018).

360 San Mauro, D. et al. Life-history evolution and mitogenomic phylogeny of caecilian amphibians. Molecular phylogenetics and evolution 73, 177-189 (2014).

361 Santana, D. J. et al. Calls and tadpoles of the species of Lysapsus (Anura, Hylidae, Pseudae). Amphibia-Reptilia 34, 201-215 (2013).

362 Santos, E. M. d. & Amorim, F. O. d. Parental care behaviour in Leptodactylus natalensis (Amphibia, Anura, Leptodactylidae). Iheringia. Série Zoologia 96, 491-494 (2006).

363 Santos-Pereira, M., Milani, D., Barata-Bittencourt, L. F., Iapp, T. M. & Rocha, C. F. D. Anuran species of the Salto Morato Nature Reserve in Paraná, southern Brazil: review of the species list. Check List 12, 1907 (2016).

364 Sartorius, S. S. & Rosen, P. C. Breeding phenology of the lowland leopard frog (Rana yavapaiensis): implications for conservation and ecology. The Southwestern Naturalist 45, 267-273 (2000).

365 Savage, J. M. The amphibians and reptiles of Costa Rica: a herpetofauna between two continents, between two seas. (University of Chicago Press, 2002).

366 Scheld, S. et al. Larval morphology and development of the Malagasy frog Mantidactylus betsileanus. Salamandra 49, 186-200 (2013).

367 Schiesari, L., Gordo, M. & Hödl, W. Treeholes as calling, breeding, and developmental sites for the Amazonian canopy frog, Phrynohyas resinifictrix (Hylidae). Copeia 2003, 263-272 (2003).

368 Schiesari, L. C., Grillitsch, B. & Vogl, C. Comparative morphology of phytotelmonous and pond-dwelling larvae of four neotropical treefrog species (Anura, Hylidae, Osteocephalus oophagus, Osteocephalus taurinus, Phrynohyas resinifictrix, Phrynohyas venulosa). Alytes 13, 109-139 (1996).

369 Schlüter, A. Reproduction and tadpole of Edalorhina perezi (Amphibia, Leptodactylidae). Studies on Neotropical Fauna and Environment 25, 49-56 (1990).

370 Schlüter, A., Löttker, P. & Mebert, K. Use of an active nest of the leaf cutter ant Atta cephalotes (Hymenoptera: Formicidae) as a breeding site of Lithodytes lineatus (Anura: Leptodactylidae). Herpetology Notes 2, 101-105 (2009).

371 Schulze, A. & Jansen, M. One species, two strategies? Oviposition site variation in a member of the Leptodactylus pentadactylus group (Amphibia: Anura: Leptodactylidae). Studies on Neotropical Fauna and Environment 47, 183-191 (2012).

372 Schweiger, S., Naumann, B., Larson, J. G., Möckel, L. & Müller, H. Direct development in African squeaker frogs (Anura: Arthroleptidae: Arthroleptis) reveals a mosaic of derived and plesiomorphic characters. Organisms Diversity & Evolution 17, 693-707 (2017).

373 Segev, O., Andreone, F., Pala, R., Tessa, G. & Vences, M. Reproductive phenology of the tomato frog, Dyscophus antongili, in an urban pond of Madagascar's east coast. Acta Herpetologica 7, 331-340 (2012).

374 Seshadri, K. S., Gururaja, K. V. & Bickford, D. P. Breeding in bamboo: a novel anuran reproductive strategy discovered in Rhacophorid frogs of the Western Ghats, India. Biological Journal of the Linnean Society 114, 1-11 (2015).

375 Sever, D., Hamlett, W., Slabach, R., Stephenson, B. & Verrell, P. in Reproductive Biology and Phylogeny of Anura (ed Barrie GM Jamieson) 319-341 (Science Publishers, Inc., 2003).

376 Seymour, R. S. & Roberts, J. D. Embryonic respiration and oxygen distribution in foamy and nonfoamy egg masses of the frog Limnodynastes tasmaniensis. Physiological Zoology 64, 1322-1340 (1991).

377 Shahriza, S., Ibrahim, J. & Shahrul Anuar, M. S. Reproductive parameters of Chalcorana labialis (Anura: Ranidae) from Peninsular Malaysia. Sains Malaysiana 45, 535-539 (2016).

378 Sheridan, J. A. & Ocock, J. F. Parental care in Chiromantis hansenae (Anura: Rhacophoridae). Copeia 2008, 733-736 (2008).

379 Silva, W. R., Giaretta, A. A. & Facure, K. G. On the natural history of the South American pepper frog, Leptodactylus labyrinthicus (Spix, 1824)(Anura: Leptodactylidae). Journal of Natural History 39, 555-566 (2005).

380 Silva, W. R. d. & Giaretta, A. A. On the natural history of Leptodactylus syphax with comments on the evolution of reproductive features in the L. pentadactylus species group (Anura, Leptodactylidae). Journal of Natural History 43, 191-203 (2009).

381 Simon, M. P. The ecology of parental care in a terrestrial breeding frog from New Guinea. Behavioral Ecology and Sociobiology 14, 61-67 (1983).

382 Souza, J. R. D., Kaefer, I. L. & Lima, A. P. The peculiar breeding biology of the Amazonian frog Allobates subfolionidificans (Aromobatidae). Anais da Academia Brasileira de Ciências 89, 885-893 (2017).

383 Sparreboom, M. & Ota, H. Notes on the life-history and reproductive behaviour of Cynops ensicauda (Amphibia: Salamandridae). Herpetological journal 5, 310-315 (1995).

384 Sparreboom, M., Xie, F. & Fei, L. Reproductive behaviour of the Chinhai salamander (Echinotriton chinhaiensis)(Caudata: Salamandridae). Amphibia-Reptilia 22, 309-320 (2001).

385 Sridhar, V. V. & Bickford, D. Oviposition site selection in the Malayan Giant Frog (Limnonectes blythii) in Singapore: conservation implications. Asian Herpetological Research 6, 184-188 (2015).

386 Stark, G. & Meiri, S. Cold and dark captivity: Drivers of amphibian longevity. Global ecology and biogeography 27, 1384-1397 (2018).

387 Stevens, R. A. A new tree-frog from Malawi (Hyperoliinae, Amphibia). African Zoology 6, 313-320 (1971).

388 Stuart, B. L. New frog records from Laos. Herpetological Review 36, 473-479 (2005).

389 Summers, K. & Earn, D. J. D. The cost of polygyny and the evolution of female care in poison frogs. Biological Journal of the Linnean Society 66, 515-538 (1999).

390 Summers, K., McKeon, C. S. & Heying, H. The evolution of parental care and egg size: a comparative analysis in frogs. Proceedings of the Royal Society B 273, 687-692 (2006).

391 Summers, K., McKeon, C. S., Heying, H., Hall, J. & Patrick, W. Social and environmental influences on egg size evolution in frogs. Journal of Zoology 271, 225-232 (2007).

392 Summers, K. & Tumulty, J. in Sexual Selection: Perspectives and Models from the Neotropics (eds Regina H Macedo & Glauco Machado) 289-320 (Academic Press, 2014).

393 Summers, K., Weigt, L. A., Boag, P. & Bermingham, E. The evolution of female parental care in poison frogs of the genus Dendrobates: evidence from mitochondrial DNA sequences. Herpetologica 55, 254-270 (1999).

394 Tabassum, F. et al. Abundance and breeding of the common skittering frog (Euphlyctis cyanophlyctis) and bull frog (Hoplobatrachus tigerinus) at Rawal Lake, Islamabad, Pakistan. Asian Herpetological Research 2, 245-250 (2011).

395 Takahashi, M. K., Okada, S. & Fukuda, Y. From embryos to larvae: seven‐month‐long paternal care by male Japanese giant salamander. Journal of Zoology 302, 24-31 (2017).

396 Tanner, W. W. Additional comments on the nesting behavior of Batrachoseps wrighti (Bishop). The Great Basin Naturalist 59, 387-389 (1999).

397 Telford, S. R. & Dyson, M. L. The effect of rainfall on interclutch interval in painted reed frogs (Hyperolius marmoratus). Copeia 1990, 644-648 (1990).

398 Telles, D. O. C., Vaz, S. A. F. & Menin, M. Reproductive biology, size and diet of Hypsiboas cinerascens (Anura: Hylidae) in two urban forest fragments in Central Amazonia, Brazil. Phyllomedusa 12, 69-76 (2013).

399 Tessa, G., Mattioli, F., Mercurio, V. & Andreone, F. Egg numbers and fecundity traits in nine species of Mantella poison frogs from arid grasslands and rainforests of Madagascar (Anura: Mantellidae). Madagascar Conservation & Development 4, 113-119 (2009).

400 Thibaudeau, G. & Altig, R. in Tadpoles: the biology of anuran larvae (eds Roy W McDiarmid & Ronald Altig) 170-188 (University of Chicago Press, 1999).

401 Thompson, E. L., Gates, J. E. & Taylor, G. J. Distribution and breeding habitat selection of the Jefferson Salamander, Ambystoma jeffersonianum, in Maryland. Journal of Herpetology 14, 113-120 (1980).

402 Toledo, L. F., Guimarães, L. D. A., Lima, L. P., Bastos, R. P. & Haddad, C. F. B. Notes on courtship, egg-laying site, and defensive behavior of Epipedobates flavopictus (Anura, Dendrobatidae) from two mountain ranges of central and southeastern Brazil. Phyllomedusa: Journal of Herpetology 3, 145-147 (2004).

403 Tóth, Z., Hoi, H. & Hettyey, A. Intraspecific variation in the egg-wrapping behaviour of female smooth newts, Lissotriton vulgaris. Amphibia-Reptilia 32, 77-82 (2011).

404 Townsend, D. S. in Contributions to West Indian herpetology: a tribute to Albert Schwartz (eds Robert Powell & Robert W Henderson) 229-239 (Society for the Study of Amphibians and Reptiles, 1996).

405 Townsend, D. S., Stewart, M. M. & Pough, F. H. Male parental care and its adaptive significance in a neotropical frog. Animal Behaviour 32, 421-431 (1984).

406 Trochet, A. et al. A database of life-history traits of European amphibians. Biodiversity Data Journal, e4123 (2014).

407 Trueb, L. Systematic relationships of neotropical horned frogs, genus Hemiphractus (Anura: Hylidae). Occasional papers of the Museum of Natural History, the University of Kansas 29, 1-60 (1974).

408 Trueb, L. & Massemin, D. The osteology and relationships of Pipa aspera (Amphibia: Anura: Pipidae), with notes on its natural history in French Guiana. Amphibia-Reptilia 22, 33-54 (2001).

409 Tsuji, H. & Kawamichi, T. Breeding habitats of a stream-breeding toad, Bufo torrenticola, in an Asian mountain torrent. Journal of Herpetology 30, 451-454 (1996).

410 Tsuji, H. & Kawamichi, T. Field observations of the spawning behavior of stream toads, Bufo Torrenticola. Journal of Herpetology 32, 34-40 (1998).

411 Tyler, M. J. & Crook, G. A. Frogs of the Magela Creek system. Vol. Technical Memorandium 19 41 (Australian Government Publishing Service, 1987).

412 Tyler, M. J., Davies, M. & Martin, A. A. Biology, morphology and distribution of the Australian fossorial frog Cyclorana cryptotis (Anura: Hylidae). Copeia 1982, 260-264 (1982).

413 Úbeda, C. A. & Nuñez, J. J. New parental care behaviours in two telmatobiine genera from temperate Patagonian forests: Batrachyla and Eupsophus (Anura: Leptodactylidae). Amphibia-Reptilia 27, 441-444 (2006).

414 Utsunomiya, Y., Utsunomiya, T. & Katsuren, S. Some ecological observations of Rana ishikawae, a rare frog endemic to the Ryukyu Islands. Proceedings of the Japan Academy, Series B 55, 233-237 (1979).

415 Vacher, J.-P. et al. Cryptic diversity in Amazonian frogs: Integrative taxonomy of the genus Anomaloglossus (Amphibia: Anura: Aromobatidae) reveals a unique case of diversification within the Guiana Shield. Molecular Phylogenetics and Evolution 112, 158-173 (2017).

416 Vaira, M. Breeding biology of the leaf frog, Phyllomedusa boliviana (Anura, Hylidae). Amphibia-Reptilia 22, 421-429 (2001).

417 Vaira, M. Annual variation of breeding patterns of the toad, Melanophryniscus rubriventris (Vellard, 1947). Amphibia-Reptilia 26, 193-199 (2005).

418 Van Dijk, D. E. Parental care in Hemisus (Anura: Hemisotidae). African Zoology 32, 56-57 (1997).

419 Van Wijngaarden, R. & Bolaños, F. Parental care in Dendrobates granuliferus (Anura: Dendrobatidae), with a description of the tadpole. Journal of Herpetology 26, 102-105 (1992).

420 Vassilieva, A. B., Galoyan, E. A. & Poyarkov Jr, N. A. Rhacophorus vampyrus (Anura: Rhacophoridae) reproductive biology: A new type of oophagous tadpole in Asian treefrogs. Journal of Herpetology 47, 607-614 (2013).

421 Vences, M. & De la Riva, I. Mantidactylus majori (NCN). Male egg guarding. Herpetological Review 36, 435-436 (2005).

422 Vera Candioti, M. F., Nuñez, J. J. & Úbeda, C. Development of the nidicolous tadpoles of Eupsophus emiliopugini (Anura: Cycloramphidae) until metamorphosis, with comments on systematic relationships of the species and its endotrophic developmental mode. Acta Zoologica 92, 27-45 (2011).

423 Verdade, V. K. & Rodrigues, M. T. A new species of Cycloramphus (Anura, Leptodactylidae) from the Atlantic Forest, Brazil. Herpetologica 59, 513-518 (2003).

424 Voituron, Y., De Fraipont, M., Issartel, J., Guillaume, O. & Clobert, J. Extreme lifespan of the human fish (Proteus anguinus): a challenge for ageing mechanisms. Biology Letters 7, 105-107 (2011).

425 Von May, R., Donnelly, M. A. & Summers, K. The tadpole of the bamboo–breeding poison frog Ranitomeya biolat (Anura: Dendrobatidae). Zootaxa 1857, 66-68 (2008).

426 Von May, R., Medina-Müller, M., Donnelly, M. A. & Summers, K. Breeding-site selection by the poison frog Ranitomeya biolat in Amazonian bamboo forests: an experimental approach. Canadian Journal of Zoology 87, 453-464 (2009).

427 Vonesh, J. R. Dipteran predation on the arboreal eggs of four Hyperolius frog species in western Uganda. Copeia 2000, 560-566 (2000).

428 Vrcibradic, D., Teixeira, R. L. & Borges-Junior, V. N. T. Sexual dimorphism, reproduction and diet of the casque-headed treefrog Itapotihyla langsdorffii (Hylidae: Lophiohylini). Journal of Natural History 43, 2245-2256 (2009).

429 Wake, M. H. in Reproductive biology of South American vertebrates (ed WC Hamlett) 112-120 (Springer, 1992).

430 Wake, M. H. Fetal adaptations for viviparity in Amphibians. Journal of Morphology 276, 941-960 (2015).

431 Wake, M. H. & Dickie, R. Oviduct structure and function and reproductive modes in amphibians. Journal of Experimental Zoology 282, 477-506 (1998).

432 Waldram, M. Breeding biology of Ranitomeya biolat in the Tambopata region of Amazonian Peru. Journal of Herpetology 42, 232-237 (2008).

433 Wassersug, R. J., Frogner, K. J. & Inger, R. F. Adaptations for life in tree holes by Rhacophorid tadpoles from Thailand. Journal of Herpetology 15, 41-52 (1981).

434 Weigandt, M., Úbeda, C. A. & Diaz, M. The larva of Pleurodema bufoninum Bell, 1843, with comments on its biology and on the egg strings (Anura, Leptodactylidae). Amphibia-Reptilia 25, 429-437 (2004).

435 Wells, K. D. The ecology and behavior of Amphibians. 1148 (University of Chicago Press, 2007).

436 Wells, K. D. & Bard, K. M. Parental behavior of an aquatic-breeding tropical frog, Leptodactylus bolivianus. Journal of Herpetology 22, 361-364 (1988).

437 Weygoldt, P. Evolution of parental care in dart poison frogs (Amphibia: Anura: Dendrobatidae). Journal of Zoological Systematics and Evolutionary Research 25, 51-67 (1987).

438 Wickramasinghe, D. D., Oseen, K. L., Kotagama, S. W. & Wassersug, R. J. The terrestrial breeding biology of the ranid rock frog Nannophrys ceylonensis. Behaviour 141, 899-913 (2004).

439 Wiens, J. J., Bonett, R. M. & Chippindale, P. T. Ontogeny discombobulates phylogeny: paedomorphosis and higher-level salamander relationships. Systematic Biology 54, 91-110 (2005).

440 Wiens, J. J., Kuczynski, C. A., Duellman, W. E. & Reeder, T. W. Loss and re‐evolution of complex life cycles in marsupial frogs: does ancestral trait reconstruction mislead? Evolution 61, 1886-1899 (2007).

441 Wilkinson, M. et al. One hundred million years of skin feeding? Extended parental care in a Neotropical caecilian (Amphibia: Gymnophiona). Biology Letters 4, 358-361 (2008).

442 Wilkinson, M. & Nussbaum, R. A. Caecilian viviparity and amniote origins. Journal of Natural History 32, 1403-1409 (1998).

443 Wilkinson, M., Sherratt, E., Starace, F. & Gower, D. J. A new species of skin-feeding caecilian and the first report of reproductive mode in Microcaecilia (Amphibia: Gymnophiona: Siphonopidae). PLoS One 8, e57756 (2013).

444 Willaert, B. et al. A unique mating strategy without physical contact during fertilization in Bombay Night Frogs (Nyctibatrachus humayuni) with the description of a new form of amplexus and female call. PeerJ 4, e2117 (2016).

445 Williams, S. R. Comparative reproduction of the endemic New Mexico plethodontid salamanders, Plethodon neomexicanus and Aneides hardii (Amphibia, Urodela, Plethodontidae). Journal of Herpetology 12, 471-476 (1978).

446 Wogel, H., Abrunhosa, P. A. & Pombal Jr, J. P. Breeding behaviour and mating success of Phyllomedusa rohdei (Anura, Hylidae) in south‐eastern Brazil. Journal of Natural History 39, 2035-2045 (2005).

447 Wolf, S. A case of male egg guarding behaviour in a stream-dwelling frog, Mantidactylus (Ochthomantis) sp. (Anura: Mantellidae), from northeastern Madagascar. Salamandra 49, 45-47 (2013).

448 Wu, H.-C., Lin, C.-F., Yeh, T.-C. & Lue, K.-Y. Life history of the spot-legged tree frog Polypedates megacephalus in captivity. Taiwan J. Biodivers. 12, 177-186 (2010).

449 Xie, F. et al. Breeding migration and oviposition of the Chinhai salamander, Echinotriton chinhaiensis. Herpetological Journal 10, 111-118 (2000).

450 Zachariah, A., Abraham, R. K., Das, S., Jayan, K. C. & Altig, R. A detailed account of the reproductive strategy and developmental stages of Nasikabatrachus sahyadrensis (Anura: Nasikabatrachidae), the only extant member of an archaic frog lineage. Zootaxa 3510, 53-64 (2012).

451 Zamudio, K. R., Bell, R. C., Nali, R. C., Haddad, C. F. B. & Prado, C. P. A. Polyandry, predation, and the evolution of frog reproductive modes. The American Naturalist 188, S41-S61 (2016).

452 Zheng, Y., Deng, D., Li, S. & Fu, J. Aspects of the breeding biology of the Omei mustache toad (Leptobrachium boringii): polygamy and paternal care. Amphibia-Reptilia 31, 183-194 (2010).

453 Zimkus, B. M., Lawson, L., Loader, S. P. & Hanken, J. Terrestrialization, miniaturization and rates of diversification in African puddle frogs (Anura: Phrynobatrachidae). PLoS One 7, e35118 (2012).

454 Zina, J. & Haddad, C. F. Ecology and reproductive biology of two species of Aplastodiscus (Anura: Hylidae) in the Atlantic forest, Brazil. Journal of Natural History 40, 1831-1840 (2006).

455 Zina, J., Silva, G. R., Loebmann, D. & Orrico, V. G. D. The recognition of Dendropsophus minusculus (Rivero, 1971)(Hylidae, Dendropsophini) as a highly polymorphic, multi-domain distributed species. Brazilian Journal of Biology 74, S146-S153 (2014).

456 Zweifel, R. G. Results of the Archbold Expeditions. No. 72. Microhylid frogs from New Guinea, with descriptions of new species. American Museum Novitates 1766, 1-49 (1956).

457 Zweifel, R. G. Australian frogs of the family Microhylidae. Bulletin of the American Museum of Natural History 182, 265-388 (1985).

458 Zweifel, R. G. Partition of the Australopapuan microhylid frog genus Sphenophryne with descriptions of new species. Bulletin of the American Museum of Natural History 68, 1-130 (2000).
