# Supplementary material for: Terrestrial reproduction and parental care drive rapid evolution in the trade-off between offspring size and number across amphibians
Source: PLoS Biol. 2022 Jan 4;20(1):e3001495. doi: 10.1371/journal.pbio.3001495 (PMC8726499; doi:10.1371/journal.pbio.3001495)
Supplement: Attachment — Submitted filename: Response_FINAL.pdf [file pbio.3001495.s011.pdf]

REVIEWERS' COMMENTS:

Reviewer #1:

This work examines macroevolutionary patterns in egg size, egg number and forms of parental care in amphibians. This analysis seeks to extend a series of comparative analyses already specifically on this group.

The authors argue that "Surprisingly, despite much research, it is still debated which selective pressures drive evolutionary changes in the offspring size-number tradeoff" and then cite an older theory paper and studies mostly focused on amphibians. The problem here is that, with a very strong focus on the amphibian literature, the authors seem to overestimated the novelty of their own work and underestimated the state of knowledge in the field more generally. For example, Lim et al. 2014 is only mentioned in passing but is a more taxonomically comprehensive exploration of ideas explored here (and controls for body size). Similarly, the idea and observation of systematic correlations among offspring size, developmental mode and fecundity goes back to at least Thorson (1936,1950), who showed strong covariance among all three for a range of marine invertebrate taxa - this work is not mentioned. This is just one example, the fact is that offspring size-number covariances have been explored repeatedly across a broad range of taxa. Plants similarly are neglected though seed size number trade-offs have been studied extensively. All in all, this work is a little tunnel visioned, focused on one group, which means the work is not placed in the appropriate context.

There seems to be a misunderstanding here regarding the objective of our study. In fact, we agree with the reviewer that there is a very large body of literature on the tradeoff between offspring size and number, which has been studied in virtually every taxonomic group. In our study we treat the existence of this tradeoff as a proven fact and the foundation of our study (please see Page 3, Lines 2-9 of the revised manuscript). In other words, we are not interested in demonstrating that there is a tradeoff between offspring size and number - we fully expect this relationship to be present in our data. Likewise, we expect allometric effects on both offspring size and offspring number as these have been repeatedly shown. For these reasons, we cite only a few papers that demonstrate the existence of the tradeoff. Specifically, we favour two reviews – one of which also discusses plants (Dani and Kodandaramaiah 2017, ref. 7 in the revised manuscript, in addition to Ronget et al.'s review on mammals and birds, Ref. 6) – and a meta-analysis that summarises the relationship between offspring size and number (Lim et al. 2014, ref. 8).

We consider the study by Lim et al. (2014) a very useful paper that summarises existing evidence since it is a meta-analysis across metazoa of the offspring size-number tradeoff *within-species* while controlling for allometry. However, Lim et al (2014) do *not* investigate the role of parental care *nor* ecology on the tradeoff, and neither care nor ecology are mentioned in their paper. Therefore, Lim et al (2014) helps us build the foundation of our own work, i.e. the evidence that there is a tradeoff between offspring size and number as well as an allometric effect on both, within and across species. Lim et al (2014) however *do not answer the question we pose in our study*: the relative importance of environmental conditions and different forms of care in altering the tradeoff.

The original aspect of our study is investigating how each of the many different forms of parental care and ecological traits influence the offspring size-number tradeoff (P3.L9-11; P3.L16-18; P4.L2-7). Furthermore, we do so not simply by showing patterns of covariation between traits as previous studies but, importantly, by testing the prediction that, if parental care and ecological traits select for (i.e. cause) changes in the tradeoff, this should result in higher rates of phenotypic evolution in

offspring size and/or number (P3.L19-P4.L2; P17.L15-P18.L3). To our knowledge, no study to date has asked this question in any system nor has used methods that allow going beyond correlation in comparative studies, including those cited by this Reviewer.

Amphibians are an ideal group for this study because they exhibit a huge diversity in parental care forms and in ecological traits related to reproduction (P7.L5-20 and Table 1 in the revised ms). Conversely, marine invertebrates exhibit a limited number of parental care strategies, mostly viviparity or brooding, and no marine group equals the diversity of parental care forms found in amphibians. In addition, many marine invertebrate taxa are poorly studied, hence lack key data to test the questions in our study, and their phylogenies are scarce and limited to a relatively few species. For these reasons, marine invertebrates are not suitable taxa in which to investigate the questions we ask in our study. Indeed, the marine invertebrate study cited by Reviewer 1 show only that 'species with viviparity or a high degree of brood protection' produce few eggs, 'species with large eggs or a primitive type of brood protection' produce an intermediate number of eggs, and 'species with small eggs and a long pelagic planktoptrophic larval life' produce many eggs (Figure 1 in Thorson 1950, n=53 species). Thorson's (1950) study does not account for phylogeny and allometry, the fact that egg size and egg number are traded off against each other, the sample size is limited, and the approach is purely correlational.

Instead, the extensive knowledge on amphibian life history traits, parental care strategies and reproductive ecology, coupled with large scale and reliable phylogenies including hundreds of species, make amphibians uniquely suitable for testing the questions we ask in our manuscript. Furthermore, we employ a cutting-edge approach that, by explicitly incorporating rates of phenotypic evolution in statistical models, can get closer to identifying causation in large scale comparative studies than other commonly used phylogenetic methods.

We appreciate that the need to make the above points clearer. Therefore, in response to the Reviewer's and other Reviewers' comments, we have revised the Introduction extensively to better explain where the novelty and originality of our study and conclusions lie (P3.L7-P6.L12; P16.L11-18), and we now clarify that the tradeoff between offspring size and number has been 'repeatedly' found in many taxa (P3.L8).

The authors then explore 16 different life history predictors and how they affect (co)variance in offspring size and fecundity. My concern here is with such an extensive list of predictors, all categorical, it's unclear how much covariance occurs among predictors and yet many of these predictors are highly correlated. Covariance among predictors leads to less reliable estimates of their effects, this is the problem with a laundry list of categorical predictors. What's more, including 16 predictors in the absence of formal explorations of their covariances means that interpreting their effects is problematic. Overall, with such a long list of predictors, I am sorry to say I have insufficient confidence in the analyses. I suggest the authors consolidate their predictors. Doing so would also reduce the impression that this is simply a pattern hunting expedition - many of justifications for the inclusions of the predictors are weak and insufficiently motivated by formal theory.

Overall, I think these are useful data for a well studied group but I don't think there's much that's new here and it doesn't really change our understanding of the topic beyond what has already been done. I think it's essential that the authors consolidate their predictor list according to the covariances among them and re-analyse their data accordingly.

The Reviewer makes an excellent point and we acknowledge that we should have reassured our readers that the covariance between the variables included in our study is low and not problematic. In response to the Reviewer's comment, we present in the revised ms a new table of the 'Variance

Inflation Factor' (VIF) for our full models (all variables) for egg and clutch size in the SI of the revised manuscript (P21.L16-24; new Table S7). VIFs quantify the extent of covariation between predictors in a given model and VIF scores greater than 5 indicate possibly problematic levels of collinearity, while greater than 10 very strong collinearity (Quinn & Keough 2002 *Experimental design and data analysis for biologists*, Cambridge Univ. Press; Ref. 81 in the revised ms). VIF scores in all our models are 2.5 or below, demonstrating that multicollinearity is not an issue in our analyses. Please also note that we computed VIFs without phylogeny; this is a conservative approach because it is well established that the inclusion of phylogeny weakens the strength of association between variables, hence VIFs in phylogenetic context are expected to be lower than when phylogeny is not considered. Please note that all the raw data used in the analyses is visually depicted on the phylogeny in Figure 3a.

We note that our sample size is 805 species (<u>none</u> with missing data), hence well beyond what is adequate to estimate effects of 16 predictor variables. Furthermore, after standard model simplification, our 'reduced' models contain 9 predictors for both egg and clutch size. Overall, given the lack of problematic multicollinearity between our predictor variables and large sample size, we are confident that our analyses are appropriate and justifiable.

Finally, with regard to the theoretical justification for inclusion of all our categorical predictor variables, we note that the inclusion is justified by current theory, empirical studies and earlier reviews, as we summarise below:

- Parental care (P4.L18-25): it has long been suggested that the evolution of parental care shift investment towards larger eggs in smaller clutches, and this is proposed by several theoretical models, which we cite in our study (Sargent et al 1987; Shine 1978, 1989; in addition we now cite also Klug and Bosnall 2010, Royle et al 2012, Pike et al 2016, Wilson 1975; Refs 13, 48-53 in our revised ms). This pattern was already proposed in early models that treated 'care' as one other form of parental investment (e.g. Trivers 1972 Parental investment and sexual selection). There are however no specific predictions as to how strongly each care form should determine an increase in egg size and decrease in clutch size and how different forms of care compare, simply because nobody before has asked this question. Thus, while theoretical models and empirical studies find that costs and benefits of caring may differ between parents or other carers, there is no specific and systematic theory that has investigated the question of whether and how different forms of care should alter the tradeoff, from which we could derive behaviour-specific and sex-specific predictions. Indeed, this represents a major gap in knowledge that our study begins to address, further demonstrating the novelty and originality of our study.
- Offspring habitat (P4.L8-17): a large body of literature, including theoretical models, empirical studies and reviews we cite (Kasimatis & Riginos 2016, Jorgensen et al 2011, McDiarmid 1978, Nussbaum & Schultz 1989, Reznick et al. 2001; References 18, 38-41), predicts that environmental conditions that increase offspring survival select for larger offspring. In amphibians and other taxa (e.g. fish) the shift to terrestrial reproduction (i.e. terrestrial eggs and/or larvae) is has long been viewed as a driver of larger eggs and smaller clutches because terrestrial habitats may reduce egg/larval mortality and larger eggs also have lower water loss, hence reduced risk of mortality by desiccation (McDiarmid 1978, Martin & Carter 2013, Magnusson & Hero 1991, Salthe & Mecham 1974, Touchon & Worley 2015, Seymour and Bradford 1995, Warkentin et al 2005; Refs. 39, 42-47).
- Direct development (P6.L7-10), which results in fully metamorphosed juvenile, should require more resources compared to eggs hatching as tadpoles, in order to sustain a prolonged period of development. Therefore, based on observations of few species, some suggested (but did not demonstrate nor quantified) that direct development should be associated with larger eggs and smaller clutches (Gomez-Mestre et al 2012, Callery et al 2001; Wells 2007; References 10, 65, 66).

There is currently no theoretical framework that allows us to make specific predictions on how the offspring size-number tradeoff should change by individual care type, stage of offspring development at which care is provided and by which sex, nor whether egg and clutch size should differ if eggs or tadpoles or juveniles are terrestrial. Our study demonstrates that this is a major oversight since we find that different care behaviours and adaptations alter either egg or clutch size or both in different directions and with different magnitude, and this is also dependent on the stage of offspring development at which a given care form is provided and by which parent (P2.L5-16; P10.L13-P13.L8; Figure 2, Figure 3B). We also demonstrate that direct development and terrestrial eggs increase egg size and decrease clutch size, while terrestrial larvae only decrease clutch size.

In response to this Reviewer's comments and suggestions by Reviewer 2 we have extensively revised the presentation of the hypotheses and predictions in the Introduction (P4.L8-P5.L16). Furthermore, we now state clearly in the Discussion that future theoretical models are needed that explicitly consider the diversity of care behaviour (P16.L11-18).

Reviewer #2:

General comments

This is a novel and important study that takes an innovative approach to testing whether parental care and offspring habitat characteristics drive the evolution of the trade-off between egg size and clutch size, and therefore life-histories in general, in amphibians.

Using an impressively large comparative dataset they test, and largely find support for, predictions from theory that parental care is associated with the evolution of increased egg size (and survival) and smaller clutch sizes are associated with terrestrial offspring environments.

The specific novelty here revolves around the use of Bayesian variable rates models that allows the Brownian motion approach (i.e. assuming a constant rate of evolution across the phylogeny) of standard Phylogenetic Generalized Least Squares models to be adapted to account for variation in the rate of phenotypic evolution. This allows the authors to quantify the direction and magnitude of the proposed drivers of the evolution in the focal traits, egg size and clutch size, i.e. parental care and offspring developmental environment, to identify particular branches of the phylogeny where there have been unusually high levels of phenotypic change. The reasonable assumption here is that this rate heterogeneity is likely to reflect variation in the strength of selection as a result of adaptive evolution driven by parental care and offspring environment. Hence, in contrast to standard comparative approaches this allows causation to be ascribed.

Furthermore, the approach also allows the trade-off between egg size and clutch size to be explicitly considered, which has not been done before. In addition, the impressively detailed dataset on over 800 species has lots of finer grained detail on specific forms of parental care. As a result the authors have uncovered some key insights, not least that clutch size shows more phenotypic evolution than egg size, most likely due to the greater degrees of freedom inherent in the number as opposed to the size of eggs, and that terrestrial habitats and direct development have consistently lead to the evolution of smaller clutch sizes and larger eggs but the effect of parental on these life-history traits depends on the particular form of care being considered.

In sum this is a very impressive piece of work that provides a substantial advance in terms of approach and insights. As such I do not have any major criticisms. However, I do think the framing of the work could be better in particular.

We thank the Reviewer for the very positive comments on the manuscript and the nice summary of the novelty and advance of our study.

Much is made of the safe harbour hypothesis, for example, which posits that parental care evolves to enhance egg survival in harsh environments. The authors suggest that the results largely provide support for this hypothesis based on the positive relationships between several different forms of parental care and egg size. However, there is also a positive effect of terrestrial offspring environment on egg size too. Given that terrestrial environments are deemed to be less harsh than aquatic environments (e.g. page 10 lines 10-12) this is somewhat confusing. As a result there is the potential for a mixed message. I don't think the data allow a specific test of the safe harbour hypothesis, which in any case is somewhat over simplistic. Instead I would be inclined to broaden it out to recognise that ecological factors more generally are associated with the initial evolution of parental care but subsequent acceleration of the rate of phenotypic evolution is facilitated by appropriate behavioural precursors and parent-offspring trait coadaptation (see e.g. Royle et al 2016 Current Opinion in Behavioral Sciences for a review). This would, in my opinion, better frame the work and largely avoid getting in a tangle about harshness or otherwise of environments promoting parental care.

We thank the Reviewer for these useful suggestions. We agree that safe harbour and environmental models are simplistic (indeed these models were proposed long ago) and the suggested paper by Royle et al offers a more advanced framework for the study of the evolutionary origin and maintenance of parental care. We have thus rephrased part of our Introduction accordingly particularly with regard to the questions related to parental care (P4.L8-25). However, we note that our objective is not to investigate the origin and maintenance of parental care but rather quantify the relative influence of diverse forms of parental care, environmental conditions (i.e. offspring habitat at the egg and tadpole stage) and direct development on the offspring-size-number tradeoff.

In addition, to broaden out the appeal of the paper still further it would also be good to add a little bit of text in the discussion that considers the broader potential implications of the work for other taxa such as birds where parental care is more ubiquitous but where there is less variation in forms of care than amphibians, or in fishes. Is the effect of parental care on the evolution of clutch size relative to egg size likely to be less marked in birds given the smaller variation in clutch size and the greater complexity of parental care for example?

We thank the reviewer for these useful suggestions; we have expanded the Discussion to emphasize the generality of our findings to other taxa (P17.L5-10).

Specific minor comments

Introduction
P3
The first paragraph of the introduction is big on bigging up the work up but leaves many basic Qs unanswered which is very frustrating rather than enticing for this reader in any case.

We agree that we have only provided some summary statements (see point by point below) in our first paragraph and provided citations for readers interested in following these up. Our reasoning is that in the first paragraph we aim to only briefly flag why understanding the drivers of the tradeoff is important and we are concerned that providing too much detail in this first paragraph will confuse readers with regard to the objectives of our study. However, we have included some additional, brief information following the Reviewer’s suggestions (see details point by point below).

9-10: for example?

In the revised text, we now explain that it is unclear to how strongly environmental conditions and/or parental care alter the tradeoff (P3.L9-11).

11-13: an example or two would be useful here too.

We have now expanded this and added a sentence with few examples (P3.L14-16).

16-20: there are two proposed drivers, so what is the difference in the prediction in terms of egg and clutch size if any? This sentence is overly vague and unspecified. Having read through the MS I know what you mean but it needs to be clearer up front.

21-22: it is still unclear what exactly it is about the new Bayesian approach that allows causation to be identified. Makes for a frustrating read at this stage.

We have dealt with these two comments together and have extensively revised this section (P3.L16-P4.L2). We now better explain our general approach and its novelty with respect to causation and provide more explicit predictions for egg and clutch size. However, we refrain from adding too many methodological details in the first paragraph of Introduction because how the methods advance the field with respect to identifying causation is explained later in the Introduction (P8.L6-P9.L18; Figure 1) and in details in the Methods section (P22.L1-P25.L9).

23: what, even under biparental care? Needs clarification.

Biparental care is rare in amphibians (only 2% of amphibian species have some form of biparental care across all forms of care combined; see Furness & Capellini 2019, ref. 69 in the revised ms) and so the sample sizes are not sufficient to consider biparental care a separate category in this study. Therefore, here we treat biparental care as the simultaneous expression of male and female care in the few species that exhibit it in our dataset. Please note that our study is not intended to investigate whether the tradeoff differs in species with biparental care vs uniparental care – for this question other taxa are more appropriate, e.g. mammals or birds. Indeed, we have already addressed this question in our earlier work in mammals (Capellini & West 2016, Ref. 56 in the revised ms). Instead, here we aim to investigate how individual care behaviours and adaptations (as well as offspring habitat and direct development) alter the tradeoff when compared to species lacking care. We have thus chosen amphibians as our model in this study because this group shows great diversity of care behaviours and adaptations and habitats in which the offspring (eggs and larvae) develop (P7.L5-20). We consider sex of the carer whenever the sample sizes are large enough to allow sufficient power for analysis (i.e. female or male egg attendance, tadpole transport and tadpole attendance; note that tadpole feeding and viviparity are exclusively provided by the female; see Table S2).

P4
17-21: so…? Isn't this a given if we accept that environmental conditions drive egg size evolution and there is a trade-off between egg size and clutch size?

Not necessarily - selection may instead act on clutch size, not of egg size, i.e. egg size may change <u>indirectly</u> through its tradeoff with clutch size, or not change at all (ditto for clutch size). For example, while mammal species with biparental care are more fecund, neonatal size does not differ in species with uniparental care vs those with biparental care (West & Capellini 2016, ref. 56).

Having identified that litter size is under selection has allowed us to reach the novel conclusion that biparental care evolved to enhance primarily the fitness of the parents via increased maternal fecundity, rather than the fitness of the offspring (West & Capellini 2016). Our earlier work in mammals thus shows that it is important to identify which trait is likely to be under selection.

In this manuscript we aim to identify whether the target of selection imposed by offspring habitat and different parental care forms is egg or clutch size (or both or neither!). This is not trivial because reasons as to why females should invest in larger eggs (e.g. enhance individual offspring survival) may differ from those explaining why they should invest in smaller clutches (e.g. being able to provision offspring post-hatching in a given habitat or because of space limitation in species carrying eggs or tadpoles). In contrast to early theoretical models focused exclusively on offspring size (e.g. Shine 1978, 1989; Nussbaum and Schultz 1989), we have found that selection has acted more strongly on clutch size than egg size, suggesting that conclusions of studies that exclusively focused on egg size may be premature (P2.L5-16; P10.L12-P12.L15; Figure 3B).

We acknowledge that we should make the above points clearer in the Introduction. Thus, to address the Reviewer's comment, we have inserted a new paragraph on these issues (P5.L1-16), expanded our explanation on a similar point related to changes in the tradeoff caused by selection on size that is unrelated to reproduction (e.g. predation) (P6.L1-7), and better summarised our results in the Abstract (P2.L12-16).

22: are they 'opposite' conclusions or just different/variable conclusions?

Agreed – rephrased as suggested

P5
5-7: difficult to understand sentence. Rewrite for clarity?

We have split the sentence into two and added further details to clarify (P6.L1-7).

P6
15-16: independently? How does this demonstrate causation?

In this paragraph (P7.L5-20 in the revised ms) we only aim to introduce the data, explain why the diversity of care forms and offspring habitat makes amphibians an excellent model for the aims of our study and for testing the predictions of two hypotheses on how care and environmental conditions affect the tradeoff. The following paragraph (P8.L6-P9.L18) instead provides an overview of the rationale of our approach and how it allows us to infer causation (by comparing rate heterogeneity in models with vs without predictors related to care and habitat; Figure 1) and tease apart the effects of different drivers by estimating the relative importance of each predictor in our model (i.e. the direction and magnitude of their independent effect, i.e. beta estimates and effect sizes). Hence, we have not added further details about how causation can be inferred with our approach in the paragraph on amphibians indicated by the Reviewer because this is explained in more details in the following paragraph (P8.L6-P9.L18) and in depth in the Methods section.

P7
10-16: very well explained.

Thank you!

18-20: ok, but that doesn't allow you to differentiate between PC and habitat as drivers does it? Would help to have a little further explanatory info here.

The comparison between a model with all significant predictors vs a model including only the tradeoff and allometry (exemplified in Figure 1): (i) estimates the relative contribution of the significant predictors (i.e. direction and magnitude of effects in the model with only significant predictors, Table S3b and S3d; Figure 2a and 2d) and (ii) demonstrates whether the significant predictors combined explain some of the observed rate heterogeneity in the response variable (egg or clutch size; Figure 1a vs Figure 1b for the predictions; Figure 2b vs Figure 2c for egg size; Figure 2e vs Figure 2f for clutch size). To further disentangle the effects of different predictors, we estimate the effect sizes of each significant predictor by comparing models when each predictor is present vs absent (Table S4). Thus, our models allow us to discriminate the magnitude of the selection of each driver on egg or clutch size through the estimation of direction of their effects and effect sizes and, in addition, demonstrate that these associations likely reflect causation since they are accompanied altogether by higher rates of phenotypic evolution in egg and clutch size.

Results
P8
14-19: could these be illustrated in a figure? It would be really useful if done well.

We have now added the percentage change due to the presence of a trait (as compared to the absence) on egg and clutch size in the revised Figure 3b.

21: wouldn't just saying "body size" be more accurate here than "allometry", given that was the term used in the models?

Agreed; we changed as suggested (P11.L11; P12.L11).

P9
11: by "terrestrial offspring" do you mean both eggs and larvae? It is a bit confusing to introduce a new term here.

Yes, we meant both eggs and larvae since both are significant predictors in our reduced model. We have now changed ‘offspring’ to ‘eggs and larvae’ (P12.L13) as suggested.

Discussion
P13
7-8: this is a really nice point but could you also broaden it out to make some predictions about other taxa such as birds or fish too, for example?

We thank the Reviewer for this and a similar suggestion above on expanding the scope of our Discussion; we have done so at P17.L5-10.

Methods
P15
12-14: how many fit this category? Info would be useful here, or refer to table.

20 species fit this category. This information is available in Table S2, which we now cite at this point in the revised ms (P19.L22-23).

P18

15: wow. That's a lot of iterations. Why so many?

The length of the chain depends on the complexity of the statistical models, the phylogenetic distribution of the data, and the number of species. We set up our models in MCMC to ensure that our posterior distributions showed convergence, good mixing and independence of subsequent samples from the chain (P23.L15-17), which can be achieved by the number of iterations and sampling period we used (P23.L12-13). There is no disadvantage of running chains of this nature for many iterations.

25: is direct development a binary variable then?

P19
1: terrestrial eggs and larvae also binary variables?

We dealt with these last two comments together as they are similar. Yes, all variables except egg size, clutch size and body size, are binary (Table S2, see title). We now make this clearer in the caption of Figure 3A and at P18.L8-9, P18.L17-18; P19.L4-5; P.19.L22-23.

Figure 2 - for a) and d) it would be useful to have "egg size" and "clutch size" respectively at the top of the posterior distributions to make it easier to see what is what.

Thank you for the suggestion, we have added these labels.

Figure 3 - a) there is crazy amounts of stuff going on here. It is very difficult to make much sense of it without assistance (e.g. highlighting key areas or changing the scale for some of the variables) or simplification. The figure looks good at a distance but it is too complicated in my opinion. b) this is very nice and simple and well explained on the other hand….

We thank the reviewer for their comment on Figure 3b and we do agree that Figure 3a conveys a lot of information and that it takes effort to take it in. However, we believe that it is also essential that we provide readers with a visual representation of the magnitude and complexity of the raw data on which all analyses were conducted. We feel this is particularly important considering our point that such complexity, ignored in most previous studies, needs to be fully considered if we are to make major advances in the field of parental care evolution and life history theory.

Table S2 - some of these variables have very small sample sizes e.g. tadpole traits, so it would be worth noting a word of caution in the results at an appropriate juncture to help interpret the effect sizes, which are quite large even for non-significant results.

The reviewer is correct that the number of species exhibiting some traits at the tadpole and juvenile stage was low (although please note that the total sample size of species is the same for all variables; n=805; Table S2). Several of these variables dropped out soon in our model simplification procedure. For completion here we note that some of the predictors that dropped out of our models had however large number of species exhibiting a trait (e.g. male and female egg attendance in the analyses of clutch size). We follow standard practice and do not interpret non-significant results as these may be caused either by a true lack of any biological effect or by low number of species in which those traits are present.

Table S5 - it would be good to have a brief summary in the main text at an appropriate juncture of some of the key clades/species that have very high rates of evolution that are given here. It would help to ground the study and make it more interesting and accessible.

We provide the list of branches, identified through their descendants, in which rates of evolution were high (without vs with our significant predictors) in Table S5 for egg size and Table S6 for clutch size. Such branches do not necessarily correspond to named clades nor necessarily match taxonomic units, and some are branches internal to the phylogeny as shown in Figure 2. We feel that describing such complex patterns is unlikely to provide major insights, be of interest to a limited number of specialists while at the same time it would require lots of heavy text. However, we now provide the pruned tree from the phylogeny by Pyron (2014; Ref. 80 in the revised ms) which we used for the analyses and that can help interested readers identify species or branches of interest.

Reviewer #3:

In this manuscript the authors analyze the relationship between offspring size and number among 800 sampled amphibian species. They find (as do previous studies) that species that lay eggs on land (including those with direct development) have larger eggs and smaller clutch sizes. They also find that different forms of parental care (e.g. male vs. female, care of egg vs. larvae) can have different effects on egg and clutch sizes, including both increases and decreases.

The authors have assembled a relatively large dataset for this study, building on their previous studies and those of other authors. They also analyze their data using a relatively recent (2016) approach that identifies shifts in rates of phenotypic evolution and identifies the correlates of those shifts.

I think that this is a very good manuscript, and I have almost no specific criticisms to make (which is very unusual for me). On the negative side, I do not think that it is of sufficiently broad interest or novelty for a top-tier journal like PLoS Biology. Basically, it is clearly an improvement over previous studies on this relatively narrow topic, and adds nuance to their conclusions. That is certainly enough to justify its existence, but not necessarily its publication in a top-tier journal. This seems more like a paper for Am. Nat., Evolution, or Proceedings B. However, I will admit that this is a relatively subjective decision.

We thank the Reviewer for the positive comments. We have taken the criticism of explaining the novelty, major advance and implications of our results very seriously and thank all Reviewers for pushing us to do better. Following Reviewers' comments, we have thus revised the text to better explain this throughout (P2.L4-5, P3.L9-11; P3.L16-P4.L7; P5.L1-16; P6.L1-12; P16.L11-18; P17.L5-10). In summary, our study is the first to consider simultaneously how different forms of parental care and reproductive ecology (offspring habitat at the egg and tadpole stage; direct development) affect the offspring size and number tradeoff, and we do so at large comparative scale (i.e. all amphibians) using cutting edge methods that can get closer to causation than other commonly used phylogenetic approaches. Given our results that parental care forms can have contrasting effects, which are unexpected by theory and not previously demonstrated, we think our findings will be of interest to a wide readership, are relevant to many other taxonomic groups, and will help advance substantially both theory on the evolution of parental care and on life history strategies.

COMMENTS FROM THE ACADEMIC EDITOR [lightly edited]:

Ref 1 is the most critical reviewer. I agree with a lot of what they say. However, I think what they say is a general criticism of ALL comparative analyses. They use the maximum data available to report

correlations between traits. Thus, in some ways, they are purely descriptive exercises. They can be treated as hypothesis testing at one level if one choses to state the direction of the correlation in advance, but a post-hoc attempt to now only look at a subset of predictors would be disingenuous. The authors already know where the strongest relationships lie. That said, the ref is right that the MS can be improved by more clearly stating which predictors are highly correlated and, perhaps, picking one of several that are highly correlated as the 'representative' of a suite of correlated predictors. They are also right that the authors need to better explain why this MS is an advance over Lim et al. 2014.

We have replied to Reviewer 1's comments point by point and revised our manuscript accordingly. In brief, we demonstrate that there is no problematic multicollinearity in our data using Variance Inflation Factors. We also point out that Lim et al. (2014) use a meta-analytical approach to investigate the consistency of the tradeoffs between offspring size and number *within species*, and on their association with body size. However, their study does not include parental care nor environmental conditions, and therefore does not address how these traits influence the tradeoff. Importantly, we are not interested in demonstrating that there is a tradeoff between offspring size and number or that these covary with body size; we take these as the starting point on which we build our study and ask instead how different forms of care and offspring habitat affects the tradeoff.

Following comments from all reviewers, we have also revised the Introduction and Discussion to better explain the novelty, major advance and implications of our results. In summary, our study is the first to consider simultaneously how different forms of parental care and reproductive ecology (offspring habitat, direct development) affect the offspring size-number tradeoff across stages of offspring development (egg, larva, juvenile), and we do so at large comparative scale (i.e. all amphibians) using cutting-edge methods that can get closer to causation than other commonly used phylogenetic approaches. Given our results that parental care forms can have contrasting effects on egg and clutch size, which are unexpected by theory and not previously demonstrated, we think our findings will be of interest to a wide readership, are relevant to many other taxonomic groups, and will help advance substantially both theory on the evolution of parental care and on life history strategies.

Ref 1 is focused on the general question which they feel has been answered in marine inverts. Perhaps this is true, but it is common for people working on different taxa to want to test relationships in their group. I think the paper will be of interest to herpetologists and that is actually sufficient to justify publication. (After all lots of bird studies are published simply because someone again shows something in birds that we already know occurs in insects, likewise for marine vs terrestrial). Crucially, I do not think the marine studies cited have the equivalent 'add on' of testing how parental care moderates the offspring size-number tradeoff.

Marine invertebrates exhibit a limited number of parental care strategies, mostly viviparity and brooding, and no marine invertebrate group equals the diversity of parental forms found in amphibians. In addition, many marine invertebrate taxa are poorly studied, hence lack key data, and phylogenies are scarce and limited to few species. For these reasons, marine invertebrates are not suitable taxa in which to investigate the questions we ask in our study – namely how different care forms, offspring habitat and direct development, influence the offspring size-number tradeoff. Indeed, the marine invertebrate study cited by Reviewer 1 (Thorson 1950) shows only that 'species with viviparity or a high degree of brood protection' produce few eggs, 'species with large eggs or a primitive type of brood protection' produce an intermediate number of eggs, and 'species with small eggs and a long pelagic planktoptrophic larval life' produce many eggs (Figure 1 in Thorson 1950,

n=53 species). This study does not account for phylogeny, the fact that egg size and egg number are traded off against each other, and that both egg and clutch size may be influenced by body size.

Instead, the extensive knowledge on amphibian life history traits, parental care strategies and reproductive ecology, coupled with large scale and reliable phylogenies including hundreds of species, make amphibians uniquely suitable for testing the questions we ask in our manuscript.

In sum, I would encourage a substantive revision that takes into account the reviewers' criticisms; which is essentially the minor points of Ref 2 and the 'reduce the number of highly correlated predictors' concern of Ref 1. Ref 3 did not ask for any changes.

We thank the Academic Editor for their clear guidance and believe that we have fully addressed all Reviewers' comments in the revised manuscript.
